# Microglial Histaminergic Signaling Promotes Interleukin-10 Production and Ameliorates Motor Dysfunction in Parkinson's Disease

**DOI:** 10.14336/AD.2025.0088

**Published:** 2026-05-07

**Authors:** Yining Wang, Minglai Zhao, Lingjuan Li, Xiaoying Liu, Liqin Lang, Xin Zhang, Zengxin Qi

**Affiliations:** ^1^Department of Neurosurgery, Huashan Hospital, Shanghai Medical College, Fudan University, Shanghai, China.; ^2^Department of Neurosurgery, Third Division General Hospital, Xinjiang Production and Construction Corps, Tumushuke, Xinjiang, China.; ^3^National Center for Neurological Disorders, Shanghai, China.; ^4^Shanghai Key Laboratory of Brain Function and Restoration and Neural Regeneration, Shanghai, China.; ^5^Neurosurgical Institute of Fudan University, Shanghai, China.; ^6^Shanghai Clinical Medical Center of Neurosurgery, Shanghai, China.; ^7^State Key Laboratory of Medical Neurobiology and MOE Frontiers Center for Brain Science, School of Basic Medical Sciences and Institutes of Brain Science, Fudan University, Shanghai, China.; ^8^Department of Nursery, Huashan Hospital, Shanghai Medical College, Fudan University.

**Keywords:** Histamine, Histamine H2 receptor, Interleukin-10, Parkinson's disease, Motor dysfunction

## Abstract

Histamine functions as a neurotransmitter regulating multiple neural processes, whereas interleukin-10 (IL-10) is an anti-inflammatory cytokine with recognized neuroprotective properties. Previous research suggests that histamine can stimulate the release of various inflammatory mediators, including IL-10. However, the precise molecular mechanisms governing the interaction between histamine and IL-10, particularly their role in safeguarding dopaminergic neurons in Parkinson's disease (PD), have not been fully elucidated. The current findings suggest that, within the context of PD, histamine levels are elevated in the substantia nigra pars compacta (SNc) microglia, leading to an upregulation of IL-10 expression through activation of the H2 receptor and the downstream cAMP/PKA/p38β/CREB signaling cascade. However, the increased histamine concentration was negatively regulated by the IL-10 expression, allowing a limited increase in its concentration. Furthermore, the H2R-IL-10 pathway activation inhibited microglial activation and the production of inflammatory factors. Moreover, the H2R–IL-10 signaling axis modulated both membrane resistance and the expression of cleaved caspase-3 mRNA in dopaminergic neurons, contributing to the improvement of motor deficits in LPS-induced mouse models. These observations suggest that, in the pathological context of PD, microglia in the SNc exhibit increased production of histamine and IL-10 in a mutually regulatory manner. Elevated histamine levels further enhance IL-10 expression, which confers neuroprotection to dopaminergic neurons through its anti-inflammatory actions, ultimately alleviating motor impairments associated with PD.

## INTRODUCTION

Parkinson's disease (PD) is a prevalent neurodegenerative disorder that affects individuals in the middle and later stages of life [1-3]. The pathogenesis of PD is primarily linked to the progressive degeneration of dopaminergic neurons within the substantia nigra pars compacta (SNc), resulting in a marked reduction of dopamine levels in the striatum [4,5]. Patients with PD typically present with motor symptoms, including bradykinesia, impaired initiation of voluntary movements, resting tremor, and increased muscle rigidity [6,7]. Moreover, PD significantly compromises patients' quality of life and, in advanced stages, can be life-threatening [8]. Epidemiological estimates indicate that the global prevalence of PD among individuals aged 65 years and older reaches approximately 1%. It is recognized as the second most common neurodegenerative disorder, following Alzheimer's disease [9]. However, there is no effective cure for PD, and treatment is only symptom-based to relieve pain [10,11]. Therefore, in-depth investigations into the pathological mechanisms driving the onset and progression of PD are critically needed, alongside the development of innovative therapeutic strategies, methodologies, and pharmacological interventions to improve disease management and patient outcomes.

Several studies have indicated that the incidence and progression of PD are associated with alterations in histamine levels within the brain [12-14]. Clinical investigations have demonstrated that histamine concentrations in the SNc of the basal ganglia in PD patients can rise to approximately 201% of those observed in healthy individuals. Moreover, elevated peripheral blood histamine levels show a significant positive correlation with the severity of motor symptoms in PD [15]. Histamine has been associated with the pathological regulation of PD, specifically by modulating the inflammatory response of microglia [16,17]. However, the exact role of histamine in regulating microglia-induced neurodegeneration remains unclear and is a topic of ongoing debate [18]. On the one hand, histamine facilitates the polarization of microglia toward a pro-inflammatory phenotype by activating the H1 or H4 histamine receptors. This activation triggers the release of pro-inflammatory cytokines such as tumor necrosis factor-alpha (TNF-α), interleukin-6 (IL-6), and interleukin-1β (IL-1β). Similarly, it promotes the generation of reactive oxygen species (ROS), nitric oxide (NO), and other inflammatory mediators, contributing to the deterioration of dopaminergic neuronal integrity and survival [16,19]. On the other hand, emerging evidence indicates that histamine may exert neuroprotective effects by inhibiting lipopolysaccharide (LPS)-induced microglial activation through H2 or H3 receptor signaling. This inhibition occurs through reducing pro-inflammatory cytokine release and enhancing anti-inflammatory cytokine secretion, thus offering protection to dopaminergic neurons [20,21]. Further research is warranted to ascertain the source of histamine and elucidate its role and mechanism in PD pathogenesis.

In the brain, IL-10 is produced by activated glial cells and serves as a highly efficacious anti-inflammatory and anti-apoptotic cytokine [22,23]. The presence of an IL-10 gene promoter polymorphism has been linked with the age of onset for PD [24]. Animal studies have shown that IL-10 exerts neuroprotective effects by suppressing the release of pro-inflammatory mediators from microglia, normalizing intracellular calcium levels, restoring the expression of anti-apoptotic proteins such as Bcl-2 and Bcl-xl, and inhibiting the activation of the apoptotic protein caspase-3 [25,26]. The SNc displays a higher density of microglia compared to other brain regions, making dopaminergic neurons in this area more vulnerable to immune-mediated responses [27,28]. A study validated that IL-10 promotes anti-inflammatory and anti-apoptotic effects by inhibiting microglia activation and dopaminergic neuron apoptosis [29]. However, the precise mechanism underlying IL-10 production in PD pathogenesis and the signaling pathways through which IL-10 inhibit microglial activation and dopaminergic neuron apoptosis to alleviate motor dysfunction remain poorly understood.

This study aimed to investigate the source, concentration dynamics, and regulatory mechanisms of histamine during PD pathology. Furthermore, the role of histamine in protecting dopaminergic neurons in the SNc and its potential to alleviate motor dysfunction in PD through modulation of IL-10 production and inhibition of microglial activation were evaluated. To achieve this, a combination of microdialysis, single-cell qPCR, qPCR, mini-osmotic drug delivery, enzyme-linked immunosorbent assay (ELISA), immunofluorescence, and behavioral assessments were employed. The findings demonstrated that in the PD mouse model, both IL-10 and histamine concentrations in the SNc progressively increased throughout the disease. Moreover, histamine decarboxylase (HDC) and IL-10 mRNA expression levels were upregulated in SNc microglia. Mechanistically, histamine enhanced IL-10 production in SNc microglia in LPS-treated mice, while IL-10, in turn, regulated histamine production to maintain its optimal concentration. Moreover, histamine was found to promote IL-10 production by H2R activation on microglia, subsequently influencing the downstream coupled cAMP/PKA/p38β/CREB pathway. Furthermore, histamine suppressed microglial activation and decreased the expression of microglia-derived pro-inflammatory cytokines by activating the H2R-IL-10 signaling pathway. This mechanism effectively inhibited apoptosis in dopaminergic neurons within the SNc, increasing membrane resistance (Rm) and reducing cleaved caspase-3 (CC3) expression. Finally, these actions contributed to improving motor dysfunction in mouse models of PD.

## MATERIALS AND METHODS

### Animals

Six- to eight-week-old C57BL/6JGpt-Rosa26^tm1Cin(CAG-LSL-Cas9-tdTomato)^/Gpt (LSL-Cas9-tdTomato, strain number T002249) and C57BL/6JGpt-Cx3cr1^em1Cin(iCre)^/Gpt (Cx3cr1-Cre; strain number T006768) mice were purchased from GemPharmatech Co., Ltd (Jiangsu, China), and wild-type (WT) C57BL/6J mice of both sexes were obtained from the Experimental Animal Center of Fudan University (Shanghai, China), and all mice were housed under specific pathogen-free (SPF) conditions at the Experimental Animal Center of Nantong University. Mice were individually housed under controlled environmental conditions (22 ± 2 °C; 60 ± 5% humidity; and 12-hour light/dark cycle with lights on at 8:00 AM daily). Animals had free access to standard laboratory chows and water. Animal care and experiments were performed in accordance with the U.S. National Institutes of Health Guide for the Care and Use of Laboratory Animals (NIH Publication 85-23, revised 2011) and were approved by the Institutional Animal Care and Use Committee of Fudan University (No. 2024-HSYY-412). Every effort was made to minimize the number of animals utilized and their suffering.

The animals of either sex, with a mean weight of 20-25 g, were randomly assigned to different treatment groups. All experiments were replicated in at least one additional independent group. Behavioral tests were conducted at the same time each day (10:00 a.m.). The experimenter performing the behavioral tests was blinded to the treatment groups for all experiments. To eliminate the confounding effects of the estrous cycle on motor behavior, only male mice were utilized in behavioral tests. Furthermore, to achieve a stable motor performance in the challenging beam test and adhesive removal test, each animal was trained daily for at least 10 trials over 3 to 5 consecutive days before the tests, as previously reported [12,30]. To mitigate stress and fatigue, the animals were permitted to rest for a minimum of 5 minutes between each behavioral trial.

### Stereotactic surgeries and injections

Under deep anesthesia induced by isoflurane (4% for induction, 1.5% for maintenance), mice weighing 20-25 g were placed into a stereotaxic frame (1404, David Kopf Instruments, Tujunga, CA) for brain surgery under aseptic conditions. A 1-2 cm incision was made in the scalp to expose the skull. A small hole was drilled in the skull, and the dura mater was carefully breached to permit uninterrupted passage of the micropipette.

### Preparation of animal model of PD and pharmacological manipulation

After anesthesia induced by isoflurane (4% for induction, 1.5% for maintenance), mice weighing 20-25 g were placed into a stereotaxic frame (1404, David Kopf Instruments, Tujunga, CA) for the LPS injection in the right SNc (AP: -3.08 mm, ML: 0.9 mm, DV: 3.85 mm) according to the mouse brain atlas[31] under aseptic conditions. A total volume of 1 µl of the LPS solution (concentration 10 μg/μl, mixed with sterile normal saline; MilliporeSigma) was then infused with Hamilton syringe(s) (2.5 μl, 7632-01) controlled by a syringe pump (KDS100, KD scientific) at a rate of 50 nl/min. The cannula was left in place for 10 min before withdrawal. On day 21 post-surgery, mice were placed in a transparent glass cylinder for a 5-minute observation period to assess exploratory behaviour. Lesion assessment was conducted by calculating the ratio of contralateral forepaw touch to total forepaw touch. Only mice that met the criteria for successful model construction were included in subsequent experiments. At the conclusion of the study, TH immunostaining in the substantia nigra pars compacta was performed to identify dopaminergic depletion.For pharmacological manipulation, mice (weighing 20-25 g) were anesthetized by isoflurane (4% for induction, 1.5% for maintenance) and then mounted on a stereotaxic frame (1404, David Kopf Instruments) for brain surgery under aseptic conditions. Mini osmotic pumps (RWD Life Science Co. Ltd, Shenzhen, China) containing saline or drugs (100 μL) connected to stainless-steel guide tubes (length 10 mm, o.d. 0.5 mm, i.d. 0.3 mm) were implanted into the right SNc (AP: -3.08 mm, ML: 0.9 mm, DV: 3.85 mm) of each animal in accordance with the mouse brain atlas. The delivery rate of the mini osmotic pumps is 0.135 μl/h, which theoretically allows for continuous drug administration over a period of 30 days as previously reported. This ensures an uninterrupted intervention in the inflammatory response process. Following the implantation, the mice were kept on a heating pad until recovery and then housed individually.

### Drugs

Histamine (MilliporeSigma, St. Louis, MO, USA; 30-300 nM), the H1R agonist 2-PyEA (R&D Systems, Minneapolis, MN, USA; 30-300 nM), the standard histamine H2R selective agonist dimaprit (R&D Systems; 30-300 nM), the potent and standard H3R agonist (R)-(-)-α-Methylhistamine (RAMH) (R&D Systems; 30-300 nM), the high-affinity H4R agonist VUF 8430 (MilliporeSigma; 30-300 nM), the selective H2R antagonist (R&D Systems; 3-30 nM), the adenylate cyclase (AC) antagonist SQ 22536 (MilliporeSigma; 10-100 nM), the protein kinase A (PKA) antagonist H-89 (R&D Systems; 3-30 nM), the p38 mitogen-activated protein kinase inhibitor SB 203580 (R&D Systems; 10-100 nM), and interleukin-10 (IL-10) (R&D Systems; 1-10 ng/ml) were freshly prepared in sterilized saline solution (0.9% NaCl). Additionally, the potent and selective cAMP response element-binding protein (CREB) inhibitor 666-15 (R&D Systems, 10-100 nM) was also freshly prepared in DMSO for use in these experiments.

### In vivo microdialysis sampling

After anesthesia by isoflurane (4% for induction, 1.5% for maintenance), adult mice weighing 20-25 g were placed into a stereotaxic frame (1404, David Kopf Instruments). Guide cannulas were stereotaxically implanted into the brain to allow positioning of a CMA/7 microdialysis probe (CMA/Microdialysis, Stockholm, Sweden) with 1 mm membrane length into the right SNc (AP: -3.08 mm, ML: 0.9 mm, DV: 3.85 mm). Probe placement was verified histologically at the end of the experiment. Mice were allowed to recover in individual cages for at least 4 days. Before sample collection in freely moving mice, microdialysis probe was inserted through the guide and the probe was perfused with ACSF for 240 minutes at a flow rate of 10 μl/min. Microdialysis samples were collected in 30 min intervals using a MAB 85 fraction collector (CMA/Microdialysis) through the microdialysis probe at 0.5 μL/min using a CMA 402 syringe pump (CMA/Microdialysis). The initial 30-minute samples were excluded, and the subsequent samples were collected for the analysis of basal extracellular levels of IL-10 and histamine using a commercial ELISA.

### Analyses of histamine, IL-10 and dopamine level

The levels of histamine and IL-10 in the SNc were quantified by competitive ELISA. In summary, the tissue was homogenised in cold PBS at 4°C and a pH of 7.2. Subsequently, the supernatant or microdialysis samples were filtered through a 0.22 μm pore-size polyvinylidene difluoride filter (MilliporeSigma) to obtain a filtered solution. The concentrations of histamine, IL-10 and dopamine were determined using histamine (Cayman Chemical, Ann Arbor, MI), IL-10 (R&D Systems, Minneapolis, MN), and dopamine (Abcam, Cambridge, UK) ELISA kits, respectively, in accordance with the instructions provided by the manufacturers. The absorbance was then read at 410 nm on a spectrophotometer, and the concentration was calculated using an equation generated from a standard curve.

### qPCR on tissue punches and single-cell qPCR

qPCR on tissue punches and single-cell qPCR were conducted as previously described [32-36], For quantification, the expression level of the target gene was normalized relative to the amount of the reference gene (Gapdh), resulting in a standardized target expression value. In negative control experiments, cDNA was substituted with water. The primer sequences are summarized as follows: TNF-α (forward) 5’-AGCCCAC GTAGCAAACCACCAA-3’, (reverse) 5’-ACACCCAT TCCCTTCACAGAGCAAT-3’; IL-6 (forward) 5’-CTG CAAGAGACTTCCATCCAG-3’, (reverse) 5’-AGTGG TATAGACAGGTCTGTTGG-3’; IL-1β (forward) 5’-GC TACCTATGTCTTGCCCGT-3’, (reverse) 5’-GACCAT TGCTGTTTCCTAGG-3’.

For single-cell qPCR, following the patch-clamp recording experiment, the cytoplasm of the identified dopaminergic neurons was pipetted into a recording pipette filled with 3-5 µl of RNase-free solution and drained into a 0.2 ml PCR tube containing Single Cell Lysis/Dnase I solution using the Single Cell-to-CT Kit (Life Technologies). Reverse transcription and cDNA pre-amplification were performed on thermal cycler (Applied Biosystems, Foster City, CA) according to the kit protocol. qPCR was performed using the TaqMan Gene Expression Assay system. The TaqMan assay probes were designed by and purchased from Life Technologies as follows: Mm00456104_m1 for HDC, Mm01288386_m1 for IL-10, Mm01195085_m1 for CC3, and Mm99999915_g1 for gapdh. Conditions for the cycles followed the manufacturer’s protocol for TaqMan assays. The data were collected using the instrument’s software (Rotor-Gene software, version 6.0, Corbett Research, Sydney, Australia) and relative quantification was performed using the comparative threshold (Ct) method after determining the Ct values for reference gene (gapdh) and target genes in each sample set according to the 2^-∆∆Ct^ method. Changes in mRNA expression levels were calculated after normalization to gapdh.

### Immunohistochemistry and imaging

Mice weighing 20-25 g were deeply anaesthetized with isoflurane (4% for induction, 1.5% for maintenance) and perfused transcardially with 50 ml normal saline, followed by 100-150 ml 4% paraformaldehyde in 0.1 M phosphate buffer. Subsequently, the brain was removed, trimmed and postfixed in the same fixative for 12 h at 4 °C, and then the brain was successively cryoprotected with 20% and 30% sucrose for 24 h. Frozen coronal sections (25 μm thickness) containing the SNc was obtained by using a freezing microtome (CM1850, Leica, Germany) and mounted on gelatin-coated slides. The slices were rinsed with PBS containing 0.1% Triton X-100 and then incubated in 10% normal bovine serum in PBS containing 0.1% Triton X-100 for 30 min.

Sections were incubated overnight at 4 °C with primary antibodies: rabbit anti-Iba1 (1:500, FUJIFILM Wako Pure Chemical Corporation, Osaka, Japan, catalog 019-19741, RRID:AB_839504), or rabbit anti-tyrosine hydroxylase (1:500, MilliporeSigma; catalog AB152, RRID:AB_390204). These primary antibodies were validated for species and application (1DegreeBio and Antibody Registry). After a complete wash in PBS, the sections for single or double immunostaining were incubated in the related Alexa Fluor 488-conjugated secondary antibodies (1:2000, Thermo Fisher Scientific) for 2 h at room temperature in the dark. The slides were washed and mounted in Fluoromount-G mounting medium (Southern Biotech, Cambridge, UK). Incubations replacing the primary antiserum with control immunoglobulins and/or omitting the primary antiserum were used as negative controls. All micrographs were taken with an inverted laser scanning confocal microscopy (model SP2 TCS; Leica, Heidelberg, Germany), equipped with Plan-Apochromat ×40/0.9 NA oil, ×20/0.75 NA dry, and ×10/0.4 NA dry objective lenses. Digital images from the microscope were recorded with Leica Application Suite imaging and analysis software (Leica) and image processing was done with Photoshop (Adobe systems Inc, San Jose, CA).

The numerical density of dopaminergic neurons in the SNc was estimated through a systematic random sampling approach, whereby 3D optical dissectors were spaced at random throughout the selected brain areas and the number of neurons within each dissector was counted. Dissectors with dimensions of 80 × 80 × 30 μm were randomly selected and the number of positive cells in each dissector was quantified. The density of cells was estimated using the following formula: Nv = Q/v (dis), where "Q" represents the average number of cells counted per dissector and "v (dis)" is the volume of the dissector, which was calculated as follows: v (dis) = a [frame] × h, where "a" is the area of the frame and "h" is the dissector height. The data were represented as the number of cells per cubic millimeter. Area density was calculated by dividing the area of the microglia, microglia cell body, microglia dendrites, or dopaminergic neurons by the total area examined in the dissectors.

### Patch clamp recordings in vitro

To evaluate the Rm of SNc dopaminergic neurons, whole-cell patch clamp recordings were conducted in accordance with the previously described methodology [12,30,32,34]. Following decapitation under deep anesthesia with isoflurane (4%), the slices containing SNc from adult mice of either sex were identified according to the mouse brain atlas [31], and incubated in oxygenated artificial cerebrospinal fluid (ACSF, composition in mM: 125 NaCl, 2.5 KCl, 1.25 NaH_2_PO_4_, 1.3 MgSO_4_, 26 NaHCO_3_, 2.5 CaCl_2_ and 10 D-glucose) at 35 ± 0.5 °C for a minimum of one hour. Subsequently, the slices were maintained at room temperature for approximately 30 minutes prior to the commencement of recordings. During this period, the slices were transferred to a submerged chamber for the duration of the recording session, and were continuously perfused with oxygenated ACSF at a rate of 2 ml/min, maintained at 32 ± 0.5 °C.

Whole-cell patch clamp recordings were conducted with borosilicate glass pipettes (4-6 MΩ) filled with an internal solution (composition in mM): 135 KMeSO4, 10 Na2-phosphocreatine. The internal solution consisted of (in mM): 5 KCl, 0.5 CaCl₂, 5 HEPES, 5 EGTA, 2 Mg₂ATP, 0.5 Na₃GTP, adjusted to pH 7.25 with KOH. The SNc was visualized with an Olympus BX51WI microscope (Tokyo, Japan), which was equipped with both infrared differential interference contrast (DIC) and fluorescence modes. All images were captured with a CoolSNAP cf Photometrics camera (Roper Scientific, Rochester, NY), displayed in a laboratory computer. Patch clamp recordings were acquired with an Axopatch-700B amplifier (Axon Instruments, Sunnyvale, CA) and the signals were fed into the computer through a Digidata-1440 interface (Axon Instruments) for data capture and analysis (pClamp 10.0, Axon Instruments). Recordings of whole-cell currents were lowpass filtered at 2 kHz and digitized at 10 kHz and recordings of membrane potentials were lowpass filtered at 5 kHz and digitized at 20 kHz. Neurons were maintained at a membrane potential of -60 mV and characterized through the injection of a rectangular voltage pulse (5 mV, 50 ms) to monitor the whole-cell series resistance and Rm. Neurons were excluded from the study if the series resistance was not stable or exceeded 30 MΩ. If the seal resistance exhibited a change exceeding 30% of the original value, the neuron was excluded from further analysis.

### Behavioral tests

#### Adhesive removal test

This test is regarded as an evaluation of motor initiation and execution [37]. Prior to the surgical procedure, two training trials were conducted by affixing two adhesive tapes (4 mm × 3 mm) to the plantar surface of both forelimbs simultaneously. The training trials were employed to acclimate the mice to the sensory stimuli (adhesive tapes), which served to diminish their anxiolytic responses during the testing sessions conducted post-surgery. At 28 days following the administration of either LPS or saline injections, adhesive tapes were placed on the forelimb contralateral to the lesioned side, and the time required for the animal to remove the tape from the forelimb was recorded.

#### Challenging beam test

The motor performance of a mouse model of PD was assessed using a novel beam test, which was adapted from traditional balance beam tests. The detailed procedure was previously described [38,39]. In brief, the beam was comprised of five sections (25 cm each) of decreasing width (3.5 cm to 0.5 cm in 1 cm decrements) and spanned 1 m in length. Animals were trained to traverse the length of the beam, starting at the widest section and ending at the narrowest section. Prior to testing, animals received three days of training, which was conducted without the mesh grid. On the day of the test, a mesh grid (1 cm square) of corresponding width was positioned over the beam surface, leaving a 1 cm space between the grid and the beam surface. Subsequently, the animals were filmed while traversing the grid-surfaced beam on five occasions. The videotapes were subsequently viewed and scored in slow motion to determine the number of errors, the number of steps, and the time required to traverse the beam. This was done for each of the five trials, and the median data across the five trials were calculated. An error was defined as a situation in which a limb (forelimb or hindlimb) slipped through the grid and was visible between the grid and the beam surface during a forward movement. Errors were not counted if the animal was not making a forward movement or when the animal's head was oriented to the left or right of the beam.

### Histological identification

On the final day of the experimental trials, the animals were deeply anaesthetised with isoflurane (4% for induction), after which the brain was removed and fixed with 4% paraformaldehyde containing 1% potassium ferrocyanide. One week later, frozen serial coronal sections (50 μm thick) were prepared and stained with Nissl. The dark blue dots indicating injection sites were identified according to the mouse brain atlas. Data from mice in which the injection sites were deviated from the SNc were excluded from further analysis.

### Statistical analysis

All data were analyzed with SPSS 17.0 (SPSS, Chicago, IL) and presented as mean ± S.E.M. A two-tailed unpaired t-test was employed for tests conducted between two groups, while one-way or two-way analysis of variance (ANOVA) followed by Student-Newman-Keuls (SNK) post hoc testing was utilized for tests involving three or more groups, post hoc tests were conducted only when the normality test was successfully passed. A threshold of P < 0.05 was accepted as statistically significant. The significance levels of the data are indicated as follows: *P < 0.05, **P < 0.01 and ***P < 0.001, *P* > 0.05 was considered non-significant and was denoted as ns. The group size (*n*) denotes independent values as detailed in the figure legend, including the number of individual animals or the number of neurons/slice/tissue typically derived from *n* = 12 mice, and statistical analyses were conducted using these independent values. Statistical methods used were all reported in Supplemental Table 1.

## RESULTS

### A correlated elevation in histamine and IL-10 levels in SNc of LPS-treated mice

**Figure 1.**
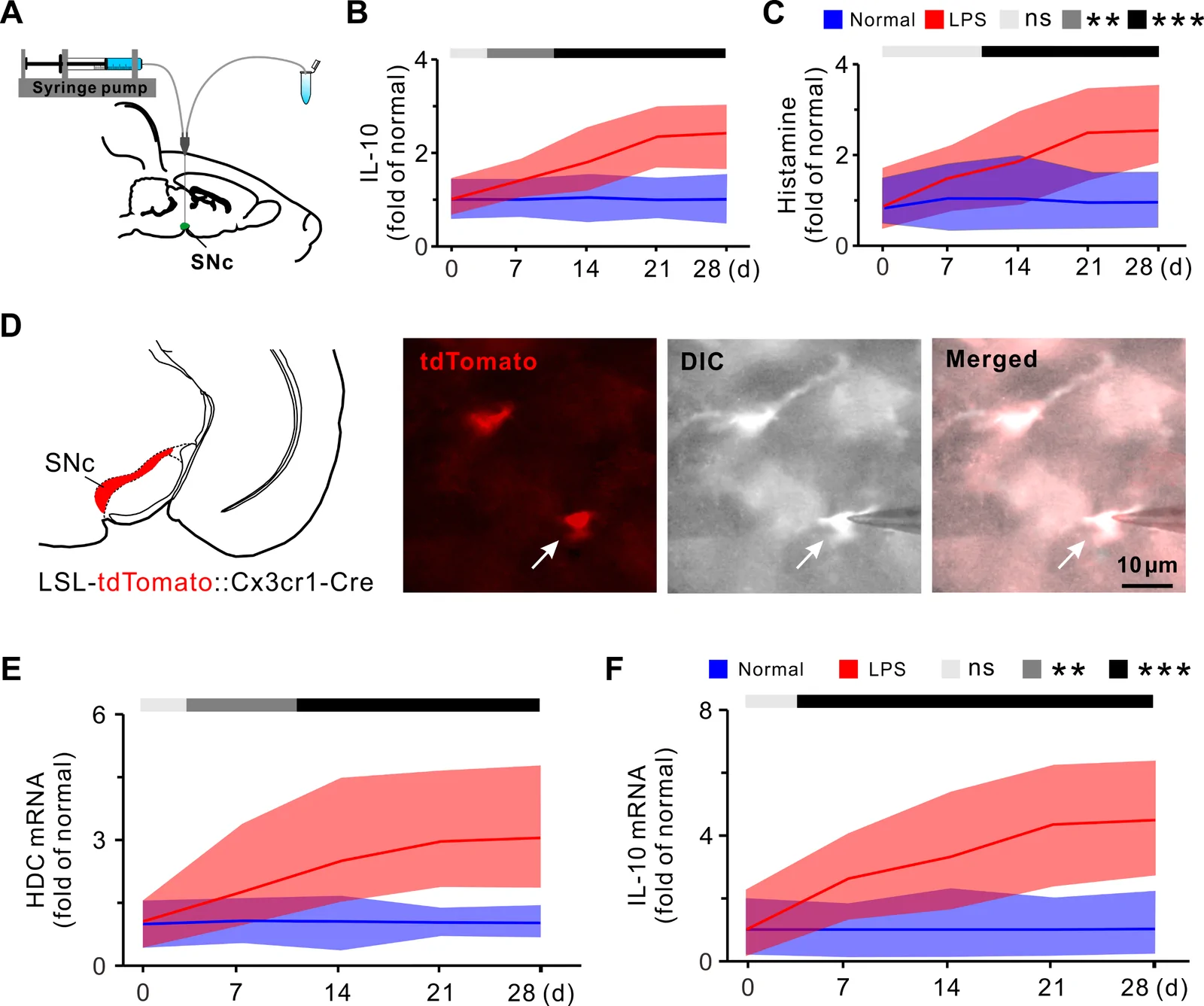
**Levels and expression of histamine and IL-10 in the SNc of LPS-treated mice.** (**A**) Schematic representation of an SNc microdialysis procedure. (**B**) IL-10 levels in the SNc of normal and LPS-treated mice (*n* = 12, two-way ANOVA and post hoc SNK test). (**C**) Histamine levels in SNc of normal and LPS-treated mice (*n* = 12 mice, two-way RM ANOVA and post hoc SNK test). (**D**) Schematic representation of microglia (arrow) in SNc of LSL-tdTomato::Cx3cr1-Cre mice labeled with tdTomato were identified under fluorescent field and selected under differential interference contrast (DIC) field for single-cell qPCR. (**E-F**) The mRNA expression of HDC and IL-10 in SNc microglia of normal and LPS-treated mice (*n* = 12 cells; two-way ANOVA and post hoc SNK test, respectively). SNc, substantia nigra pars compacta. Data are represented as mean ± SEM; ns, no statistical difference, ***P < 0.001.

Given that PD is characterized by the degeneration of dopaminergic neurons in the SNc, a mouse model of PD was utilized to evaluate IL-10 concentration in the SNc *via* microdialysis at days 0, 7, 14, 21, and 28 (Fig. 1A). The results showed a significant increase in IL-10 concentration in the SNc of LPS-treated mice throughout the progression of PD (Fig. 1B). Similarly, histamine concentrations also rose significantly during PD pathogenesis (Fig. 1C). Moreover, previous studies have suggested that microglia are capable of synthesizing and releasing histamine [40,41].

Others suggest that microglia can synthesize and release IL-10 [42,43]. However, it remains unclear whether histamine and IL-10 are released simultaneously during the progression of PD. To address this, microglia were isolated from SNc brain slices of both normal and LPS-treated LSL-tdTomato::Cx3cr1-Cre mice (Fig. 1D), and the expression of histamine decarboxylase (HDC) and IL-10 genes in microglia was assessed using single-cell qPCR. The results demonstrated a significant upregulation in the expression of HDC and IL-10 genes during PD progression (Fig. 1E-F). These findings suggest that histamine and IL-10 are co-expressed by microglia in the SNc of LPS-treated mice.

**Figure 2.**
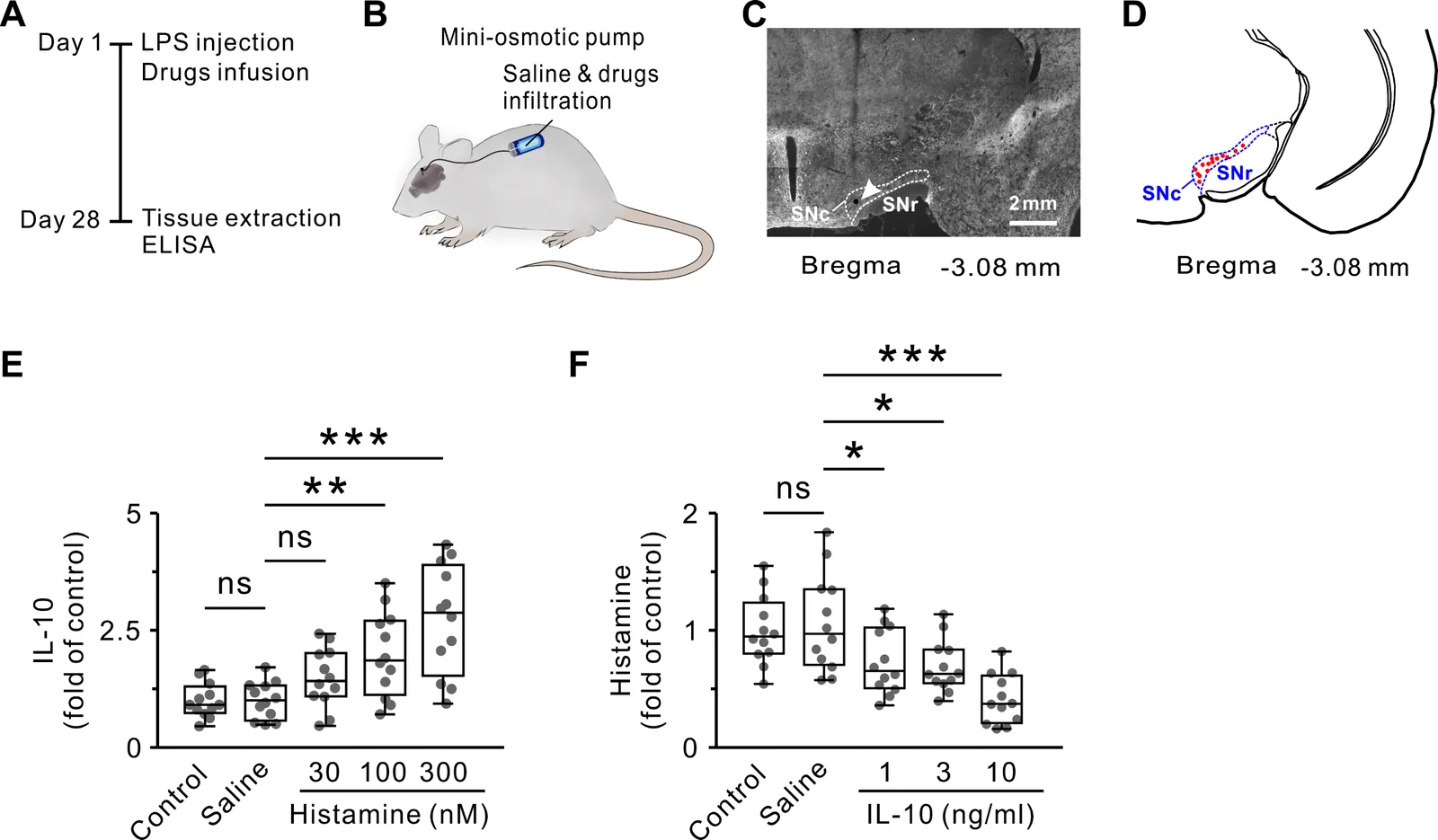
**Histamine potentiated IL-10 production on microglia of LPS-treated mice.** (**A**) Diagram of the experimental timeline. (**B**) Schematic representation of saline or histaminergic drug infiltration in SNc using a mini-osmotic pump. (**C**) Representative coronal section of mini-osmotic pump injection site of unilateral SNc (indicated by arrowheads). (**D**) Representative histological reconstruction map of unilateral SNc across 12 animals. (**E**) Infiltration of histamine to assess the production of IL-10 in LPS-treated mice (*n* = 12 mice, one-way ANOVA and post hoc SNK test, respectively). (**F**) Infiltration of IL-10 to assess the production of histamine in LPS-treated mice (*n* = 12 mice, one-way ANOVA and post hoc SNK test, respectively). Data are represented as mean ± SEM; ns, no statistical difference, **P < 0.01 and ***P < 0.001.

Previous literature has demonstrated that histamine induces the production of inflammatory mediators from microglia [19,44]. In this study, the impact of histamine on IL-10 production by microglia was examined by using a mini-osmotic pump to deliver histamine into the SNc (Fig. 2A-D). The results demonstrated that histamine infiltration (30-100 nM) into the SNc led to a dose-dependent increase in IL-10 production (Fig. 2E), suggesting that histamine may enhance IL-10 secretion by microglia in LPS-treated mice. Similarly, IL-10 (1-10 ng/mL) was also administered into the SNc *via* the mini-osmotic pump, which resulted in a dose-dependent reduction of histamine levels (Fig. 2F). Furthermore, as the concentrations of both IL-10 and histamine increased, the mRNA expression of histamine decarboxylase (HDC) and IL-10 also elevated during PD progression, reaching a plateau after 21 days (Fig. 1B-C, E-F). Based on these findings, it is hypothesized that histamine promotes IL-10 production in the SNc of LPS-treated mice, which maintains histamine concentrations at a relatively stable level by inhibiting its production.

### Histamine potentiated IL-10 production *via* cAMP/PKA/p38β/CREB signaling pathway coupled to H2R on microglia

**Figure 3.**
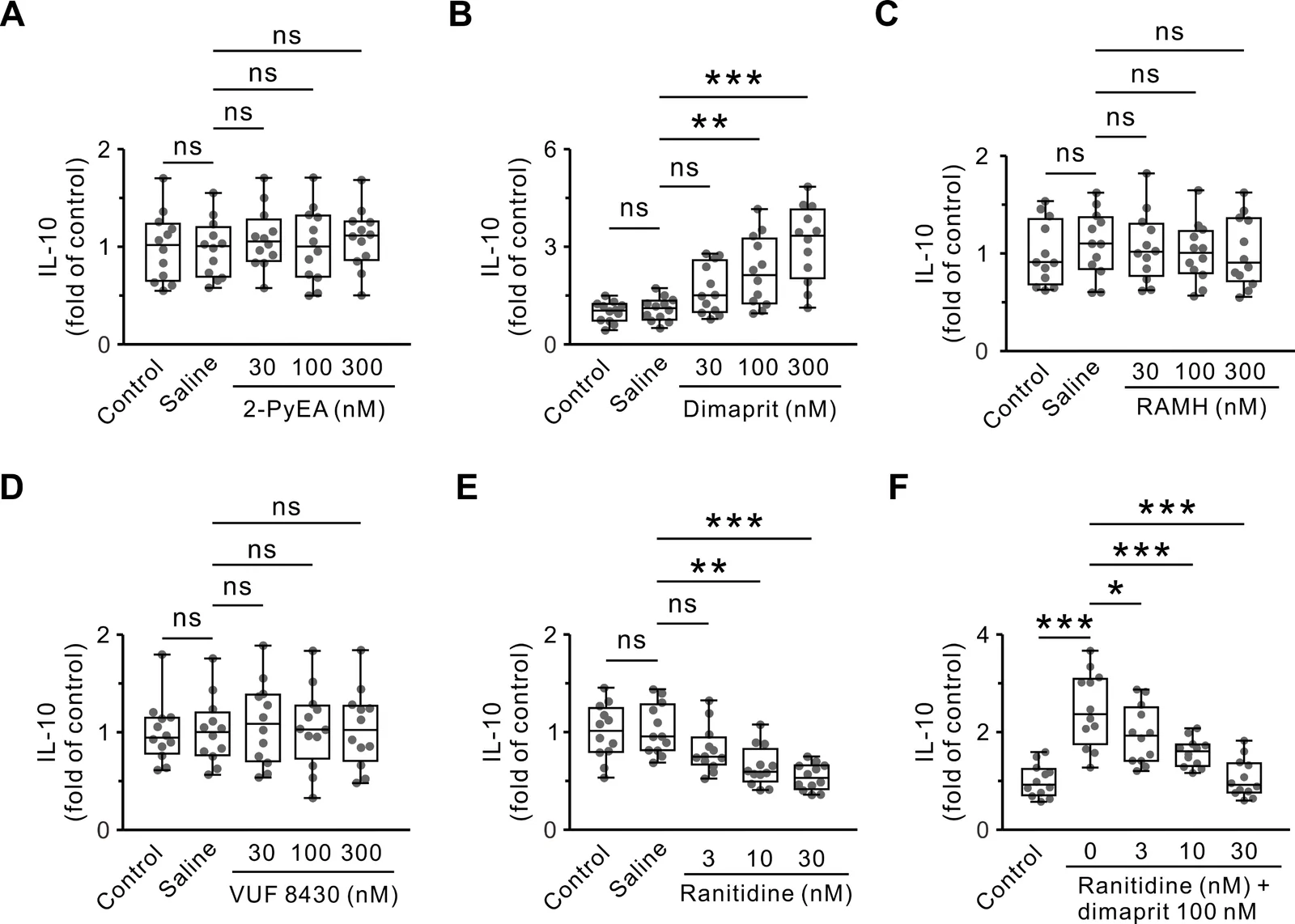
**Histamine potentiated IL-10 production *via* H2R on microglia in LPS-treated mice.** The effect of (**A**) H1R agonist 2-PyEA, (**B**) H2R agonist dimaprit, (**C**) H3R agonist RAMH, (**D**) H4R agonist VUF 8430, and (**E**) H2R antagonist ranitidine infiltration in SNc on the production of IL-10 in LPS-treated mice (*n* = 12 mice, one-way ANOVA and post hoc SNK test, respectively). (**F**) The effect of ranitidine and dimaprit co-infiltration in SNc on the production of IL-10 in LPS-treated mice (*n* = 12 mice, one-way ANOVA, and post hoc SNK test, respectively). Data are represented as mean ± SEM; ns, no statistical difference, **P < 0.01 and ***P < 0.001.

The current study further explored the mechanism through which histamine induces IL-10 production in the SNc during the progression of PD. Importantly, all four histamine receptor subtypes were found to be expressed in microglia [45]. This study first assessed which histamine receptors mediate IL-10 production from microglia. Histamine receptor antagonists or agonists were infiltrated *via* a mini-osmotic pump into the SNc. The data indicated that the selective H2 receptor agonist recapitulated the effects of histamine, dimaprit (30 - 300 nM), but not by the H1 receptor agonist 2-PyEA (30 - 100 nM), the potent H3 receptor agonist RAMH (30 - 100 nM), or the high-affinity H4 receptor agonist VUF 8430 (30 - 100 nM) (Fig. 3A-D), suggesting that the H2 receptor plays a key role in mediating these effects. Furthermore, the H2 receptor antagonist ranitidine (3 - 30 nM) inhibited IL-10 production dose-dependent (Fig. 3E), indicating that blockade of the H2 receptor prevented endogenous histamine-induced IL-10 production. The effect of dimaprit (100 nM) on IL-10 production was similarly dose-dependently inhibited by ranitidine (3 - 30 nM) (Fig. 3F). These findings highlight the critical involvement of the H2 receptor in modulating histamine's effect on IL-10 production in microglia of LPS-treated mice.

**Figure 4.**
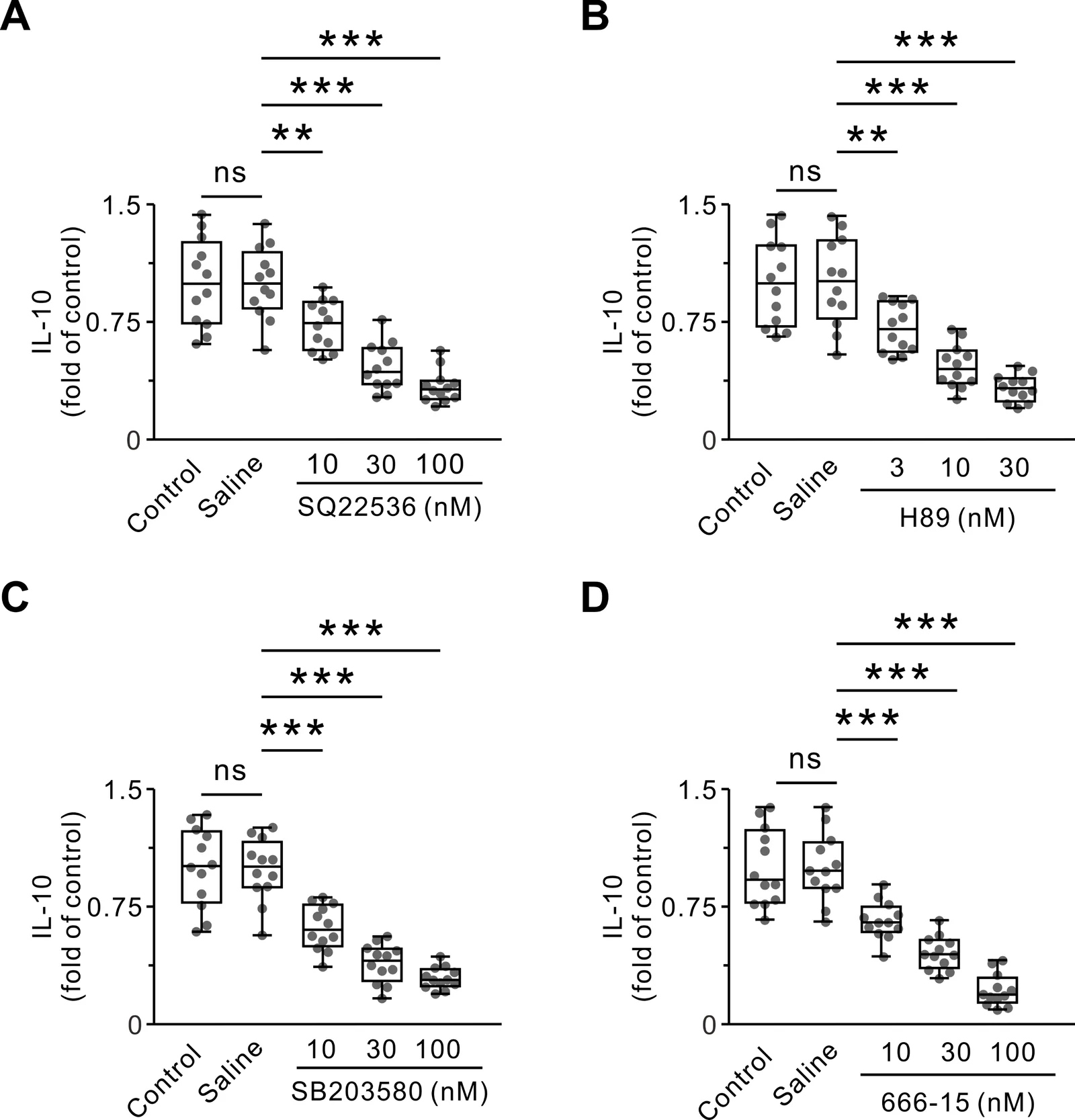
**H2R-coupled cAMP/PKA/p38β/CREB pathway regulated IL-10 production in microglia of LPS-treated mice.** (**A**) The effect of the adenylyl cyclase antagonist SQ 22536, (**B**) PKA antagonist H-89, (**C**) p38 antagonist SB 203580, and (**D**) CREB inhibitor 666-15 infiltration in SNc on IL-10 production in LPS-treated mice (*n* = 12 mice, one-way ANOVA and post hoc SNK test, respectively). Data are represented as mean ± SEM; ns, no statistical difference, **P < 0.01 and ***P < 0.001.

Previous studies have shown that increased cAMP levels accelerated IL-10 transcription [46] and enhanced the downstream cascade H2R constitutive activity coupled to Gs proteins stimulating AC and elevated intracellular cAMP, which activates protein kinase A and the transcription factor cAMP response element-binding protein (CREB) [47]. A series of experiments were conducted to determine whether the Gs/cAMP/PKA/CREB signaling pathway is involved in the action of H2R on IL-10 production. Briefly, antagonists for adenylyl cyclase (SQ 22536, 10 - 100 nM), PKA (H-89, 3 - 30 nM), p38 (SB 203580, 10 - 100 nM), and CREB (KG 501, 10 - 100 nM) were administered into the SNc. The results showed that these treatments dose-dependently reduced IL-10 production in LPS-treated mice (Fig. 4A-D), implicating the cAMP/PKA/p38β/CREB signaling pathway in this process. These findings suggest that histamine enhances IL-10 production through the cAMP/PKA/p38β/CREB pathway, which is coupled to H2R activation on microglia in LPS-treated mice.

### Pharmacological activation of the H2R-IL-10 pathway attenuated microglia activation by inhibiting the production of inflammatory factors

**Figure 5.**
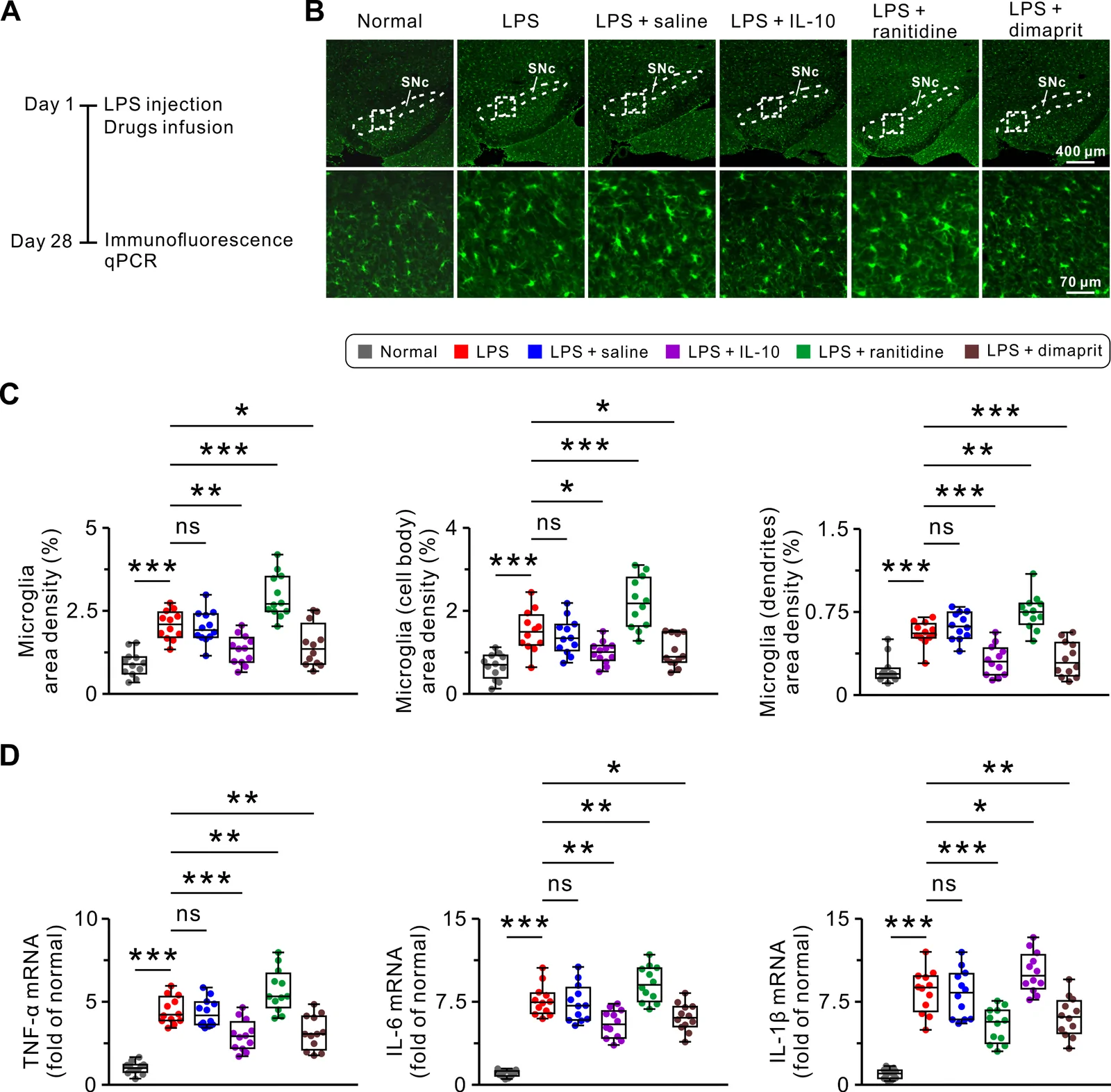
**Activation of the H2R-IL-10 pathway attenuated microglial activation by inhibiting the production of inflammatory factors.** (**A**) Diagram of the experimental procedure. (**B**) Representative images of microglia in SNc of a normal mouse, a LPS-treated mouse, and a LPS-treated mouse infiltrated with saline, IL-10, ranitidine, and dimaprit in the SNc, respectively. (**C-D**) The effect of IL-10, ranitidine, and dimaprit on the area density of microglia, its cell bodies and dendrites, as well as the expression of inflammatory factors including TNF-α, IL-6, and IL-1β (*n* = 12 slice/tissue, one-way ANOVA and post hoc SNK test, respectively). Data are represented as mean ± SEM; ns, no statistical difference, *P < 0.05, **P < 0.01 and ***P < 0.001.

In the central nervous system, microglial activation is the hallmark of neuronal lesions or degeneration, which leads to the production of pro-inflammatory cytokines [26,44]. This study investigated if the H2R-IL-10 pathway could be a therapeutic target to prevent excessive microglia activation, which promotes neurodegenerative disease in LPS-treated mice. Briefly, dimaprit, ranitidine, or IL-10 were infiltrated in SNc (Fig. 5A), and the data revealed that dimaprit mimics the IL-10-induced reduction of the area density of microglia, their cell bodies, and dendrites. In comparison, ranitidine had the opposite effects (Fig. 5B-C). These results indicated that the H2R-IL-10 signaling pathway plays a role in attenuating microglial activation. Moreover, the effects of dimaprit, ranitidine, and IL-10 on the production of pro-inflammatory cytokines by microglia were assessed using qPCR on tissue punches from the SNc. The findings showed that dimaprit mimicked the inhibitory effect of IL-10 on microglial pro-inflammatory cytokine production. In comparison, ranitidine infiltration produced the opposite effects (Fig. 5D). Overall, these data suggest that histamine exerts an inhibitory influence on microglial activation and the production of pro-inflammatory factors through the H2R-IL-10 pathway.

**Figure 6.**
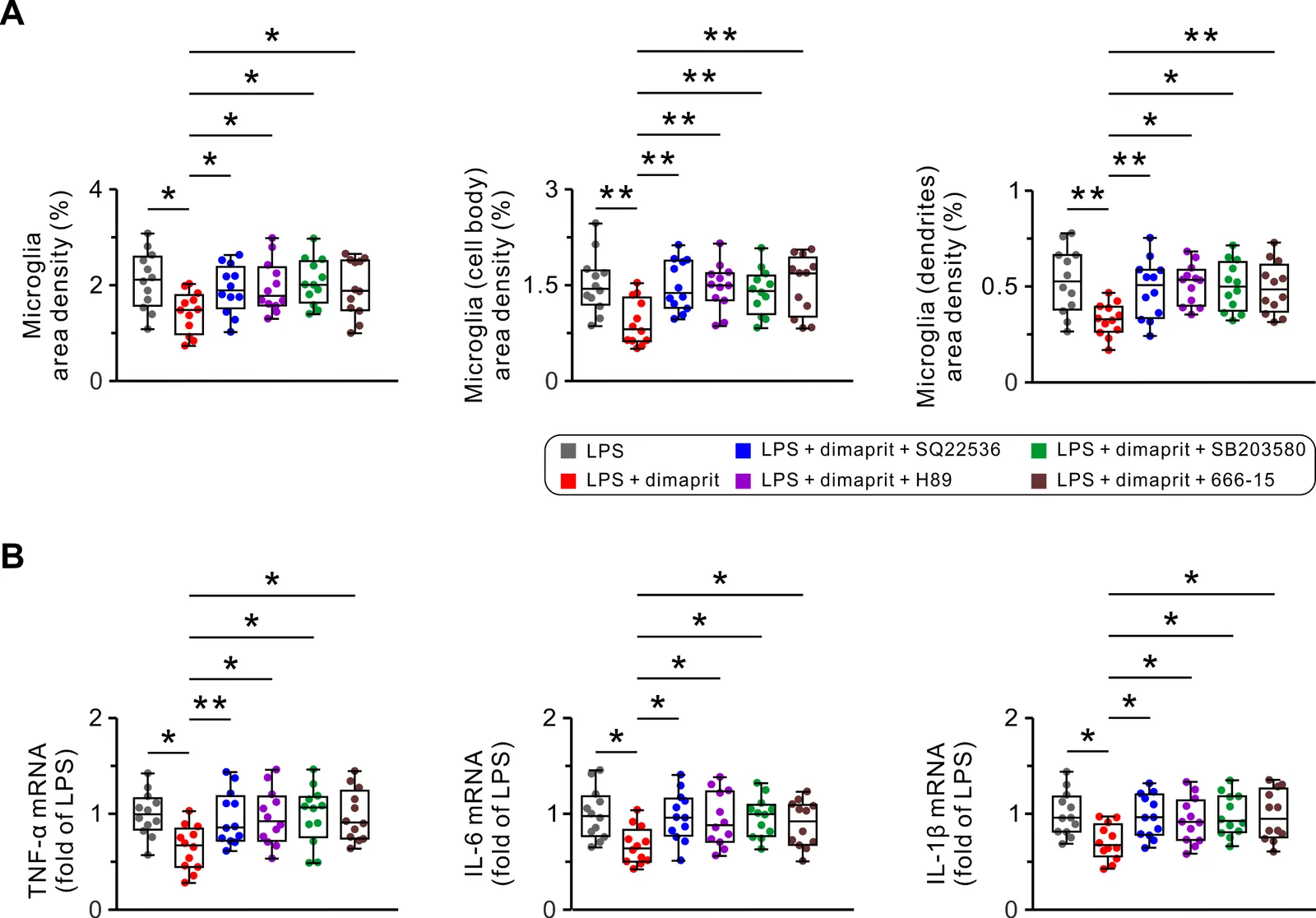
**H2R-coupled cAMP/PKA/p38β/CREB pathway regulated microglial activation and inflammatory factor release.** (**A**) The effect of SQ22356, H89, SB203580, and 666-15 on the area density of microglia, as well as its cell bodies and dendrites (*n* = 12 slice/tissue, one-way ANOVA and post hoc SNK test, respectively). (**B**) The effect of SQ22356, H89, SB203580, and 666-15 on the expression of inflammatory factors including TNF-α, IL-6, and IL-1β (*n* = 12 slice/tissue, one-way ANOVA and post hoc SNK test, respectively). Data are represented as mean ± SEM; *P < 0.05 and **P < 0.01.

This study also investigated the involvement of the H2R-coupled Gs/cAMP/PKA/CREB signaling pathway in microglial activation and the production of pro-inflammatory cytokines. Briefly, dimaprit (100 nM) was administered alone or in combination with SQ 22536 (20 nM), H-89 (10 nM), SB 203580 (50 nM), and KG 501 (25 nM) into the SNc. The results showed that SQ 22536, H-89, SB 203580, and KG 501 effectively blocked the impact of dimaprit on microglia. Moreover, these treatments inhibited the dimaprit-induced reduction in microglial area density and its cellular structures (Fig. 6A) while also suppressing the expression of pro-inflammatory cytokines such as TNF-α, IL-6, and IL-1β (Fig. 6B). These findings suggest that histamine inhibits microglial activation and pro-inflammatory cytokine production through the cAMP/PKA/p38β/CREB signaling pathway, which is coupled to the H2 receptor on microglia in the substantia nigra pars compacta of LPS-treated mice.

### The H2R-IL-10 pathway attenuates dopaminergic neuron degeneration in LPS-treated mice

**Figure 7.**
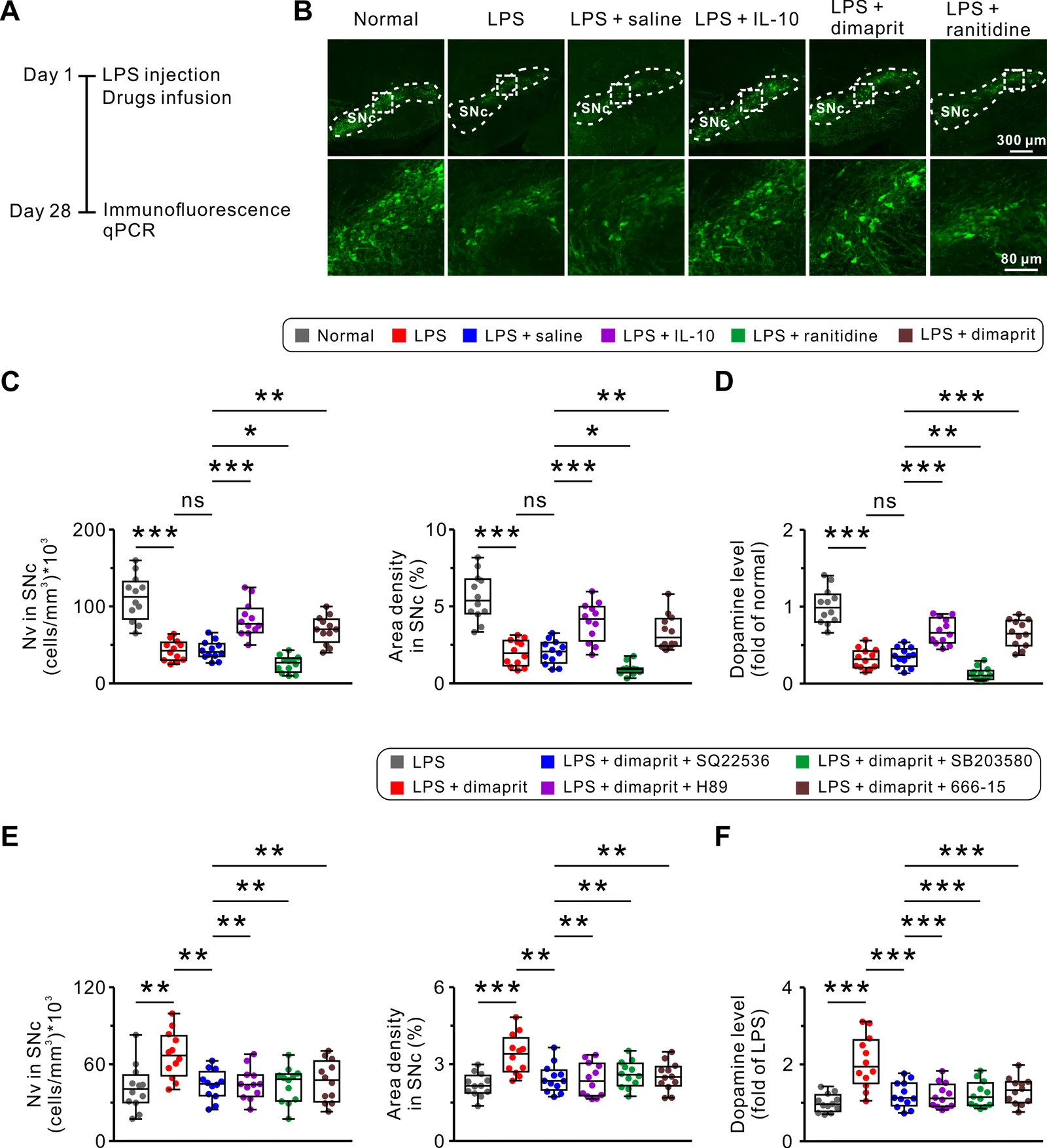
**H2R-IL-10 pathway activation attenuated dopaminergic neuron loss.** (**A**) Diagram of the experimental procedure. (**B**) Representative image of dopaminergic neurons in SNc of normal control, LPS-treated control, and LPS-treated saline, IL-10, ranitidine, and dimaprit infiltrated groups. (**C-D**) Effect of IL-10, ranitidine, and dimaprit as well as (**E-F**) SQ22356, H89, SB203580, and 666-15 on the numerical density (Nv) and area density of dopaminergic neurons, as well as the dopamine level in the striatum (*n* = 12 slice, one-way ANOVA and post hoc SNK test, respectively). Data are represented as mean ± SEM; *P < 0.05, **P < 0.01 and ***P < 0.001.

Since the degeneration of SNc dopaminergic neurons characterizes PD [4,5,12], the effect of histamine on SNc dopaminergic neuron degeneration was assessed by affecting the H2R-IL-10 signaling pathway in LPS-treated mice. Briefly, IL-10, dimaprit, and ranitidine were infiltrated in the SNc of mice after LPS treatment (Fig. 7A). On the 28th day, brain slices containing SNc were sampled, which showed that dimaprit simulated the protective effect of IL-10 on dopaminergic neurons (Fig. 7B). Moreover, administration of dimaprit or IL-10 significantly increased both the numerical and area densities of dopaminergic neurons compared to the saline injection group. However, ranitidine exhibited the opposite effect (Fig. 7C). Further analysis of striatal dopamine levels using the microdialysis technique revealed that compared to the saline-treated SNc group, administration of dimaprit and IL-10 resulted in increased striatal dopamine levels. However, ranitidine administration led to a reduction in striatal dopamine levels (Fig. 7D). Moreover, the role of the H2R downstream coupled with the cAMP/PKA/p38β/CREB signaling pathway in the degeneration of SNc dopaminergic neurons, as well as the levels of dopamine in the striatum of LPS-treated mice, was assessed. In brief, dimaprit (100 nM), both alone and in combination with SQ 22536 (20 nM), H-89 (10 nM), SB 203580 (50 nM), and KG 501 (25 nM), were administered into the substantia nigra pars compacta (SNc). The results showed that treatment with SQ 22536, H-89, SB 203580, and KG 501 effectively prevented the dimaprit-induced increase in the density and area of dopaminergic neurons, as well as attenuating the elevated dopamine concentration in the striatum of LPS-treated mice (Fig. 7E-F). These findings suggest that the observed effects are mediated through the cAMP/PKA/p38β/CREB signaling pathway. In summary, it can be inferred that histamine protects dopaminergic neurons in the SNc *via* the H2R-IL-10 signaling pathway, elevating striatal dopamine levels in LPS-treated mice.

**Figure 8.**
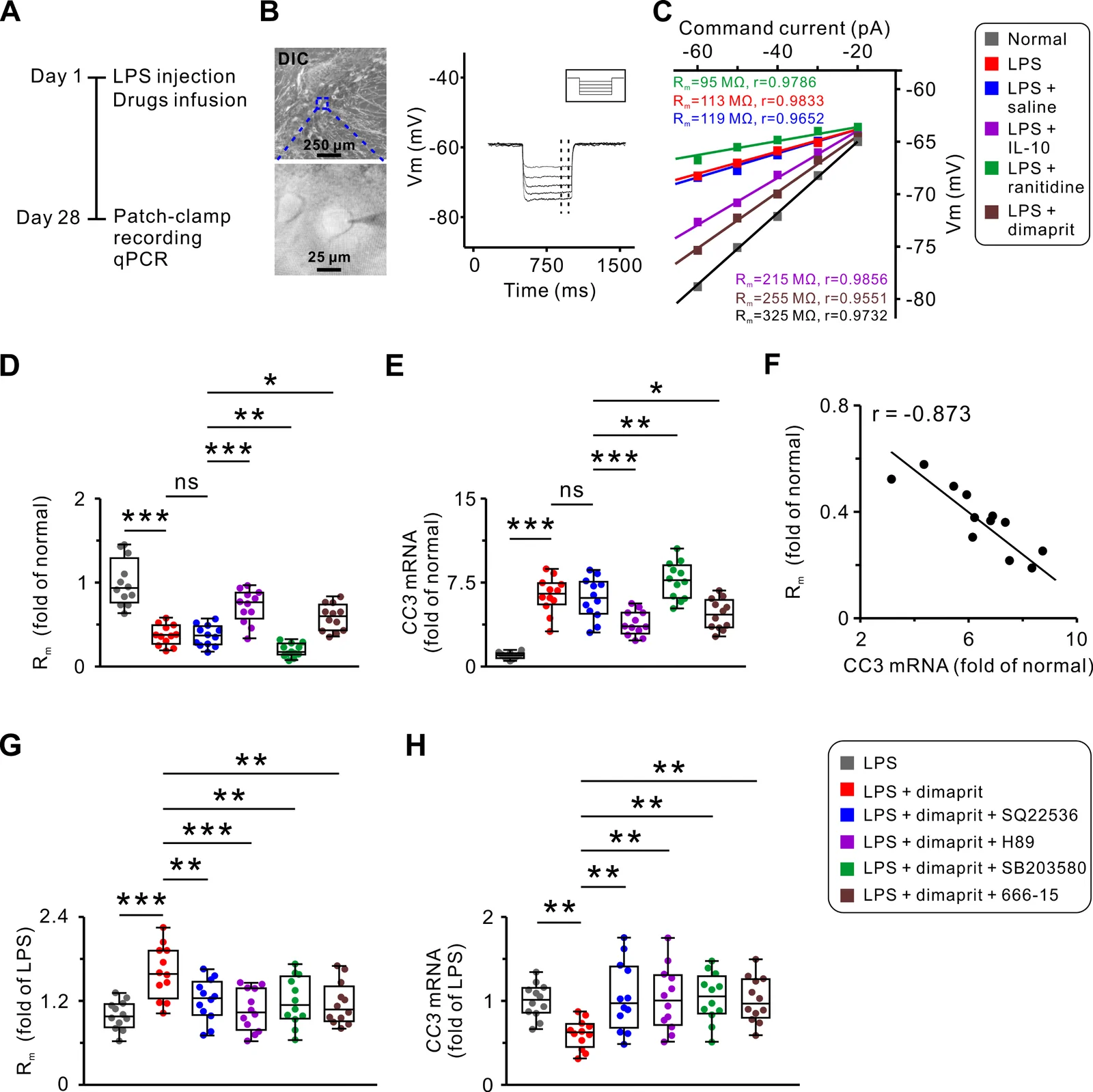
**H2R-cAMP/PKA/p38β/CREB pathway regulated the mRNA expression of Rm and CC3 in dopaminergic neurons of LPS-treated mice.** (**A**) Diagram of the experimental procedure. (**B**) Representative diagram of the Rm acquired *via* step current stimulation. The step current’s amplitudes were adjusted to the voltage between -65 and -75 mV. (**C**) Average traces between 900 and 950 ms were calculated to stand for the membrane voltage’s changes, and the I-V curves were plotted on dopaminergic neurons in SNc of normal control, LPS-treated control, and LPS-treated saline, IL-10, ranitidine, and dimaprit infiltration groups. A linear regression was applied, and r > 0.95 was considered successful. (**D**) Infiltration of IL-10, ranitidine and dimaprit on the Rm of dopaminergic neurons (*n* = 12 neurons, one-way ANOVA and post hoc SNK test, respectively). (**E**) Effect of IL-10, ranitidine, and dimaprit on CC3 mRNA expression in dopaminergic neurons (*n* = 12 neurons, one-way ANOVA and post hoc SNK test, respectively). (**F**) The Rm was negatively correlated with CC3 mRNA expression in the dopaminergic neurons of LPS-treated mice (*n* = 12 neurons, Pearson Correlation). (**G**) Effect of SQ22356, H89, SB203580, and 666-15 on the Rm and (**H**) CC3 mRNA expression in dopaminergic neurons (*n* = 12 neurons, one-way ANOVA and post hoc SNK test, respectively). Data are represented as mean ± SEM; *P < 0.05, **P < 0.01 and ***P < 0.001.

This study also investigated how histamine protects dopaminergic neurons in the SNc *via* the H2R-IL-10 pathway in LPS-treated mice. Since dopaminergic neuron apoptosis in the SNc is a key characteristic of PD and is closely linked to the Rm of neurons [48], the impact of the H2R-IL-10 signaling pathway on the Rm of dopaminergic neurons was assessed in LPS-treated mice. Briefly, the PD mouse model was induced by intraperitoneal injection of LPS, and IL-10, ranitidine, and dimaprit was subsequently administered to the SNc (Fig. 8A). After 28 days, the Rm of SNc dopaminergic neurons was examined using the patch-clamp technique (Fig. 8B). The data revealed that the Rm of SNc dopaminergic neurons was decreased in LPS-treated mice. However, sustained IL-10 and dimaprit infiltration increased the neuronal Rm, while ranitidine had the opposite effect (Fig. 8C-D). It has been observed that active CC3 facilitates apoptosis by modulating the formation of membrane blebs in the plasma membrane [49]. This study examined the expression of the *CC3* gene in dopaminergic neurons using single-cell qPCR, which revealed a significant increase in *CC3* gene expression in dopaminergic neurons of the SNc in LPS-treated mice.

In comparison, sustained administration of IL-10 and dimaprit significantly reduced *CC3* gene expression. However, prolonged administration of ranitidine produced the opposite effect, increasing *CC3* gene expression (Fig. 8E). Furthermore, the relationship between Rm and *CC3* gene expression in SNc dopaminergic neurons was assessed 14 days after LPS injection. The results demonstrated an inverse correlation between CC3 gene expression and Rm (Fig. 8F). Moreover, the association of the cAMP/PKA/p38β/CREB signaling pathway was assessed on the Rm and CC3 gene expression in LPS-treated mice. It was found that sustained infiltration of dimaprit with SQ22536, H89, SB203580, or 666-15 inhibited the dimaprit-induced increase in Rm and the decrease in CC3 expression of SNc dopaminergic neurons, respectively (Fig. 8G-H). Overall, it was observed that histamine promotes a protective effect on SNc dopaminergic neurons by enhancing Rm and reducing CC3 expression *via* the H2R-IL-10 signaling pathway.

**Figure 9.**
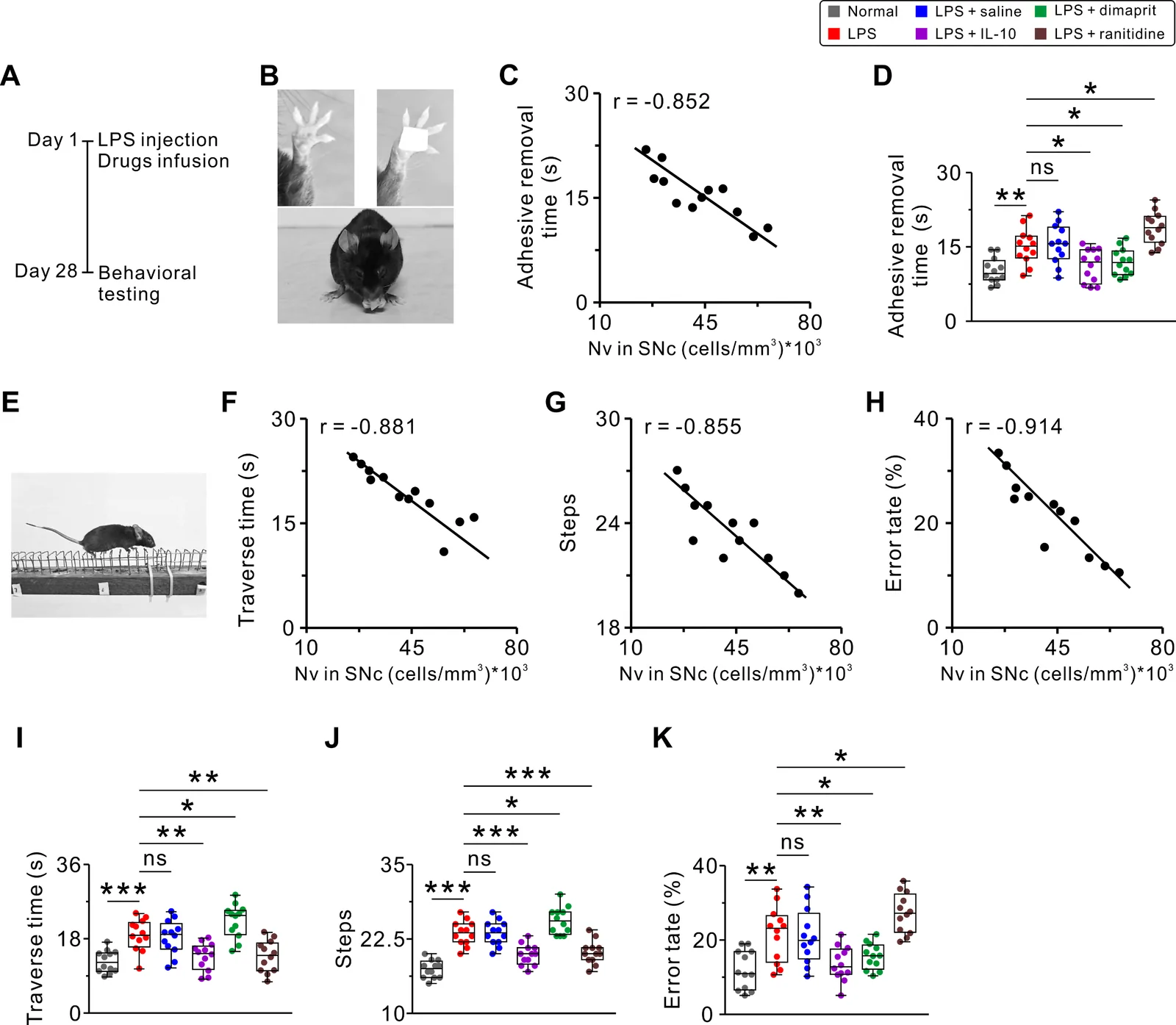
**Infusion of IL-10 and dimaprit rather than ranitidine ameliorated motor dysfunction in LPS-treated mice.** (**A**) Diagram of the experimental procedure. **(B)** Diagram of the adhesive removal test. (**C**) The numerical density of dopaminergic neurons was negatively correlated with adhesive removal time in LPS-treated mice (*n* = 12 neurons, Pearson Correlation). (**D**) Infusion of IL-10 and dimaprit, but not ranitidine, into the SNc, ameliorates motor dysfunction by shortening adhesive removal time in LPS-treated mice.**(E)** Diagram of the challenging beam test. (**F-H**) The numerical density of dopaminergic neurons was negatively correlated with the total time to traverse the beam (**F**), number of steps (**G**), and error rate (**H**) in LPS-treated mice (*n* = 12 neurons, Pearson Correlation). (**I-K**) Infusion of IL-10 and dimaprit, but not ranitidine, into the SNc ameliorated motor dysfunction by reducing the total time to traverse the beam (**I**), number of steps (**J**), and error rate (**K**) in LPS-treated mice (*n* = 12 mice, one-way ANOVA and post hoc SNK test). Data are represented as mean ± SEM; *P < 0.05, **P < 0.01 and ***P < 0.001.

### H2R-IL-10 pathway ameliorated motor dysfunction in LPS-treated mice

**Figure 10.**
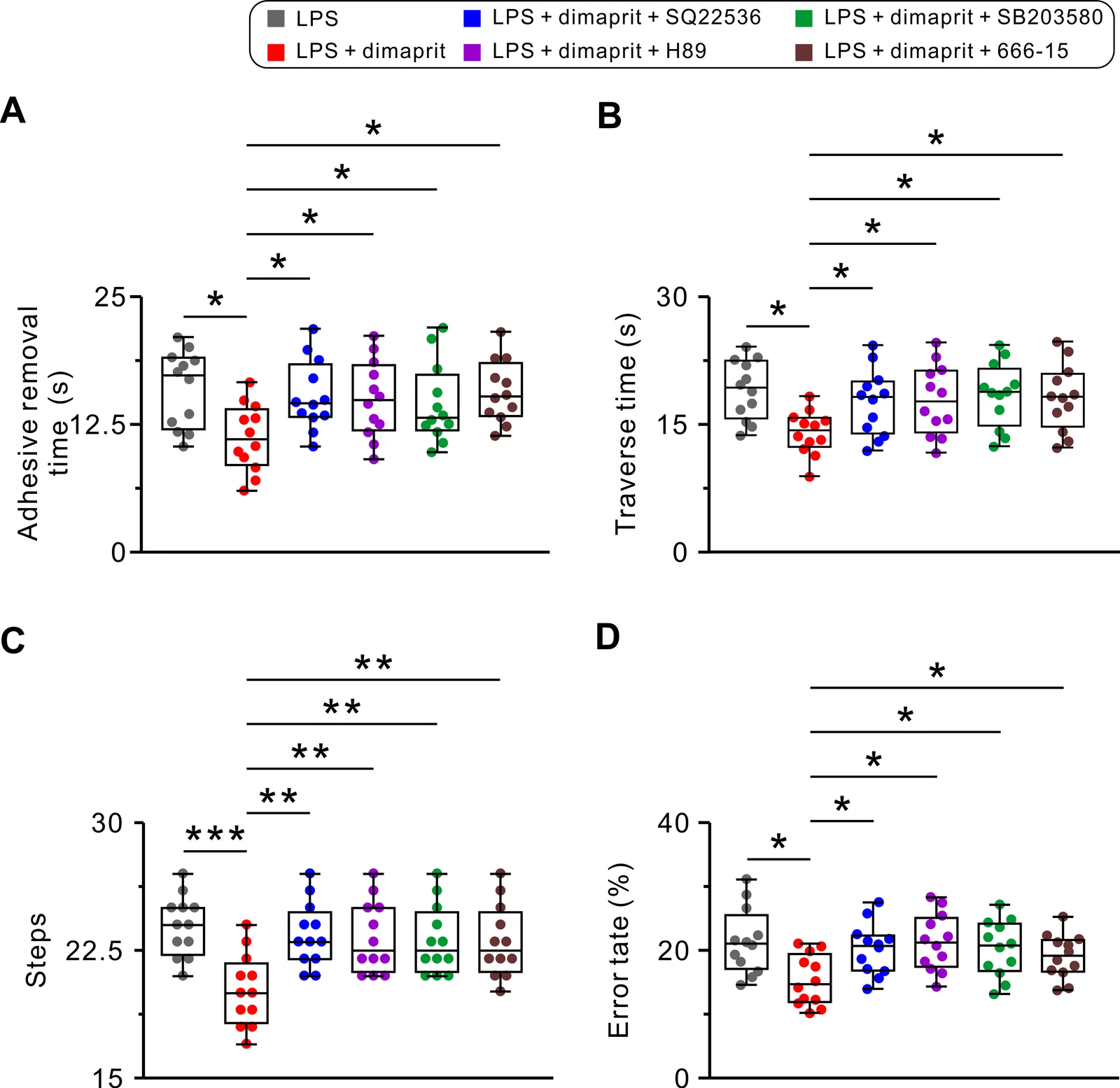
**Inhibition of the cAMP/PKA/p38β/CREB pathway prevented the amelioration of motor dysfunction by dimaprit in LPS-treated mice.** (**A-C**) Infiltration of SQ22356, H89, SB203580, and 666-15 into the SNc prevented the amelioration of dimaprit-induced motor dysfunction by increasing the total time to traverse the beam (**A**), number of steps (**B**), and error rate (**C**) in LPS-treated mice. (**D**) Infiltration of SQ22356, H89, SB203580, and 666-15 into the SNc prevented the amelioration of dimaprit-induced motor dysfunction by prolonging the adhesive removal time in LPS-treated mice (*n* = 12 mice, one-way ANOVA and post hoc SNK test). Data are represented as mean ± SEM; *P < 0.05, **P < 0.01 and ***P < 0.001.

Given that H2R-IL-10 signaling has been shown to exert a neuroprotective effect on dopaminergic neurons in the SNc, the potential of this pathway to alleviate motor dysfunction in LPS-treated mice was evaluated. Initially, a significant negative correlation was observed between the numerical density of SN neurons in LPS-treated mice and the motor behavior demonstrated by the animals during the adhesive removal test (Fig. 9A-C) and the challenging beam test (Fig. 9E-H). The LPS-treated mice were bilaterally administered IL-10, dimaprit, or ranitidine into the SNc *via* a mini-osmotic pump. The effect of IL-10, dimaprit, and ranitidine on motor dysfunction was then assessed *via* the adhesive removal test (Fig. 9D) and challenging beam test (Fig. 9I-K). The results indicated that chronic micro-infiltration of IL-10 (100 nM) and dimaprit (100 nM) effectively alleviated motor dysfunction. This was demonstrated by a reduction in the total time, number of steps, and error rate required to traverse a beam and a decreased adhesive removal time in LPS-treated mice. However, micro-infiltration of ranitidine (100 nM) exacerbated motor dysfunction, as evidenced by increased adhesive removal time and elevated total time, number of steps, and error rate to traverse the beam in LPS-treated mice. Furthermore, co-infiltration of dimaprit (100 nM) with SQ22536, H89, SB203580, or 666-15 significantly mitigated the effects of dimaprit on motor dysfunction, as reflected by an increase in adhesive removal time, total time, number of steps, and error rate to traverse the beam (Fig. 10A-D). These results indicate that histamine ameliorates motor dysfunction *via* the H2R-IL-10 signaling pathway in LPS-treated mice.

## DISCUSSION

Neuroinflammatory responses accompany the activation of microglia and the production of inflammatory factors during PD pathogenesis [50-52]. SNc contains a higher density of microglia, which renders dopaminergic neurons more susceptible to immune responses [27]. Histamine, an endogenous biogenic amine, has been observed to regulate immune responses in the brain [16]. Furthermore, it functions as a neurotransmitter and a modulator of the immune system [53,54]. IL-10 prevents inflammatory and autoimmune pathologies by modulating the pathogen's immune responses [22]. The present study demonstrated that the levels of IL-10 and histamine in the SNc and the expressions of HDC and IL-10 were significantly elevated in the LPS-treated PD mouse model. Histamine was found to stimulate IL-10 production, which, in turn, regulates histamine expression by negatively modulating its production. Furthermore, histamine promoted IL-10 production *via* the cAMP/PKA/p38β/CREB signaling pathway downstream of H2R. Moreover, histamine inhibited microglial activation in the SNc of LPS-treated mice through the H2R-IL-10 pathway, therefore exerting a protective effect on dopaminergic neurons and ultimately improving motor dysfunction in LPS-treated mice.

Cytokine family includes IL-10, IL-19, IL-20, IL-22, IL-24, IL-26, IL-28A, IL-28B, and IL-29 [22,25]. IL-10 has been studied in animal models and human disease states [42,55,56]. This study found that IL-10 production and gene expression were significantly enhanced in SNc of LPS-treated mice (Fig. 1B, F). Studies suggest that LPS can stimulate the generation of IL-10 in microglia of amyotrophic lateral sclerosis mice model [57]. Moreover, low-dose LPS can mitigate early brain injury following subarachnoid hemorrhage by increasing IL-10 production [58]. In the present study, LPS-treated mice were used as controls, and a dose-dependent increase in IL-10 concentration was observed with continuous histamine infiltration into the SNc (Fig. 2F). Based on these findings, it was inferred that the elevated IL-10 levels observed in PD pathology could be attributed to the increased histamine concentration.

Several studies have suggested that alterations in the brain histamine concentrations may play a significant role in the initiation and progression of PD [13,54]. Clinical studies have indicated that histamine levels in the SNc of PD patients can increase by up to 201%. Moreover, the degree of increase in blood histamine levels has been significantly correlated with the severity of PD symptoms [15]. The data presented here indicate that histamine levels and HDC expression increased considerably as PD progressed, with histamine concentration reaching a peak by day 21 (Fig. 1C, E). Moreover, the concentration of IL-10 and gene expression also increased with PD progression (Fig. 1B, F). Further investigations revealed that histamine increased the production of IL-10, while IL-10 suppressed the production of histamine (Fig. 2E-F). These results suggest that elevated IL-10 levels prevent an excessive rise in histamine concentration by inhibiting its production through IL-10 receptor activation, thus stabilizing histamine levels at a plateau (Fig. 1C). Whereas since histamine stimulates IL-10 production *via* histamine receptors, the stability of histamine levels also elevates IL-10 concentration to a plateau (Fig. 1B). However, the mechanism by which IL-10 inhibits histamine production remains undetermined.

It is well-established that four distinct types of histamine receptors (H1R, H2R, H3R, and H4R) exhibit a unique distribution pattern within the brain. Furthermore, these receptors are associated with different downstream signaling pathways [16,19,59]. Some studies have shown that activation of H1R, H3R, and H4R stimulates the production of inflammatory factors, such as TNF-α and IL-1β, therefore contributing to a pro-inflammatory response [16,19,60]. Other studies have suggested that the activation of H2R and H4R can inhibit inflammatory factor production, exerting an anti-inflammatory effect [19,60,61]. Therefore, the microenvironment of microglia and their activation state might be related to these differing outcomes.

Histamine exerts anti-inflammatory effects by binding to H2R and activating its downstream signaling pathways. Mechanistically, histamine interacts with H2R, inhibiting leukotriene synthesis in human neutrophils *via* cAMP-dependent protein kinase A (PKA) signaling [62]. Furthermore, glucagon-like peptide-1 receptor activation has been observed to stimulate IL-10 expression in microglia through the autocrine cAMP/PKA/p38β/CREB signaling pathway [63]. Elevated cAMP concentrations in mouse macrophages can increase IL-10 transcription, thus inhibiting inflammatory responses [46]. In this study, it was found that IL-10 production from microglia was enhanced by histamine by modulating H2R (Fig. 3) and its associated cAMP/PKA/p38β/CREB signaling pathway (Fig. 4). This suggests that H2R-mediated microglial activation induces an anti-inflammatory effect by promoting IL-10 production. The findings of this study align with previous research, which proposes that histamine acts as an anti-inflammatory agent, increasing IL-10 production through the activation of H2R and its associated downstream signaling pathways [44,64]. Since H1R, H3R, and H4R are also expressed in microglia, further investigation is required to elucidate their effect on microglia.

Numerous studies have demonstrated that neuronal apoptosis is closely associated with membrane resistance. During apoptosis, voltage-dependent anion channels in the cell membrane of hippocampal neuronal cell lines are activated, and blocking these channels can prevent the onset of apoptosis [65]. Moreover, high clinical doses of lidocaine can destroy the cell membrane, which can induce neuronal morphological changes, reduce Rm, increase membrane capacitance in a dose-dependent manner, as well as induce neuronal necrosis and apoptosis [48]. Similarly, estrogen pretreatment mitigated glutamate-induced apoptosis and preserved electro-physiological function in primary cortical neurons by enhancing Na+/capacitance levels and reducing glutamate-induced caspase-3 activation [66]. During toxin-induced neuronal apoptosis, the activation of caspase-3 is preceded by the opening of voltage-dependent anion channels on the neuronal cell membrane [67]. In this study, histamine treatment was found to alleviate motor dysfunction in LPS-treated mice by inhibiting the activation of SNc microglia and dopaminergic neuronal apoptosis and enhancing neuronal Rm *via* the H2R-IL-10 signaling pathway. These findings suggest that changes in neuronal Rm are closely linked to the onset of apoptosis. Therefore, targeting H2R and its associated downstream signaling pathways represents an effective strategy to mitigate microglial inflammatory responses in PD mouse models, increase neuronal Rm, and provide protective effects on dopaminergic neurons.

### Conclusion

This study demonstrated that histamine and IL-10 concentrations in the SNc were significantly elevated during PD pathogenesis. Histamine promoted IL-10 production, which, in turn, regulated histamine levels by inhibiting its synthesis. Furthermore, histamine increased IL-10 production through the H2R and PKA pathways downstream of H2R. Moreover, histamine mitigated microglial activation and the release of inflammatory factors *via* the H2R-IL-10 pathway. Histamine also enhanced the Rm of neurons, inhibited the apoptosis of dopaminergic neurons, and played a neuroprotective role. *In vivo* analysis also showed amelioration in motor dysfunction. These findings support the hypothesis that the H2R-IL-10 signaling pathway represents a promising therapeutic target for clinically inhibiting neuroinflammatory responses, protecting neurons, and improving motor dysfunction in PD. However, the exact mechanisms by which other histamine receptor types influence dopaminergic neurons, mainly through their interactions with microglia in PD motor dysfunction, remain poorly understood.

## Data Availability

All data supporting the findings of this study are available from the corresponding author upon reasonable request.

## References

[1] Tolosa E, Garrido A, Scholz SW, and Poewe W (2021). Challenges in the diagnosis of Parkinson's disease. Lancet Neurol, 20:385-397. 10.1016/S1474-4422(21)00030-2 PMC8185633 33894193

[2] Bhat S, Acharya UR, Hagiwara Y, Dadmehr N, and Adeli H (2018). Parkinson's disease: Cause factors, measurable indicators, and early diagnosis. Comput Biol Med, 102:234-241. 10.1016/j.compbiomed.2018.09.008 30253869

[3] De Virgilio A, Greco A, Fabbrini G, Inghilleri M, Rizzo MI, Gallo A, et al. (2016). Parkinson's disease: Autoimmunity and neuroinflammation. Autoimmun Rev, 15:1005-1011. 10.1016/j.autrev.2016.09.027 27725149

[4] Jiang Y, Qi Z, Zhu H, Shen K, Liu R, Fang C, et al. (2025). Role of the globus pallidus in motor and non-motor symptoms of Parkinson's disease. Neural Regen Res, 20:1628-1643. 10.4103/NRR.NRR-D-23-01660 PMC11688550 38845220

[5] Qi ZX, Yan Q, Fan XJ, Peng JY, Zhu HX, Jiang YM, et al. (2024). Role of HCN channels in the functions of basal ganglia and Parkinson's disease. Cell Mol Life Sci, 81:135. 10.1007/s00018-024-05163-w PMC10937777 38478096

[6] Ben-Shlomo Y, Darweesh S, Llibre-Guerra J, Marras C, San Luciano M, and Tanner C (2024). The epidemiology of Parkinson's disease. Lancet, 403:283-292. 10.1016/S0140-6736(23)01419-8 PMC11123577 38245248

[7] Chen ZC, Li GL, and Liu J (2020). Autonomic dysfunction in Parkinson's disease: Implications for pathophysiology, diagnosis, and treatment. Neurobiol Dis, 134:104700. 10.1016/j.nbd.2019.104700 31809788

[8] Wang S, Jiang Y, Yang AC, Meng FG, and Zhang JG (2025). The Expanding Burden of Neurodegenerative Diseases: An Unmet Medical and Social Need. Aging Dis, doi: 10.14336/AD.2024.1071. PMC12339136 39571158

[9] Dorsey ER, Sherer T, Okun MS, and Bloem BR (2018). The Emerging Evidence of the Parkinson Pandemic. J Parkinson Dis, 8:S3-S8. 10.3233/JPD-181474 PMC6311367 30584159

[10] Chen H, Kwong JC, Copes R, Tu K, Villeneuve PJ, van Donkelaar A, et al. (2017). Living near major roads and the incidence of dementia, Parkinson's disease, and multiple sclerosis: a population-based cohort study. Lancet, 389:718-726. 10.1016/S0140-6736(16)32399-6 28063597

[11] Feigin VL, Nichols E, Alam T, Bannick MS, Beghi E, Blake N, et al. (2019). Global, regional, and national burden of neurological disorders, 1990-2016: a systematic analysis for the Global Burden of Disease Study 2016. Lancet Neurol, 18:459-480. 10.1016/S1474-4422(18)30499-X PMC6459001 30879893

[12] Peng JY, Qi ZX, Yan Q, Fan XJ, Shen KL, Huang HW, et al. (2023). Ameliorating parkinsonian motor dysfunction by targeting histamine receptors in entopeduncular nucleus-thalamus circuitry. Proc Natl Acad Sci U S A, 120:e2216247120. 10.1073/pnas.2216247120 PMC10151461 37068253

[13] Shan L, Bao AM, and Swaab DF (2015). The human histaminergic system in neuropsychiatric disorders. Trends Neurosci, 38:167-177. 10.1016/j.tins.2014.12.008 25575625

[14] Zhu HX, Lou WW, Jiang YM, Ciobanu A, Fang CX, Liu CY, et al. (2025). Histamine Modulation of the Basal Ganglia Circuitry in the Motor Symptoms of Parkinson's Disease. Cns Neurosci Ther, 31:e70308. 10.1111/cns.70308 PMC11866051 40013534

[15] Rinne JO, Anichtchik OV, Eriksson KS, Kaslin J, Tuomisto L, Kalimo H, et al. (2002). Increased brain histamine levels in Parkinson's disease but not in multiple system atrophy. J Neurochem, 81:954-960. 10.1046/j.1471-4159.2002.00871.x 12065607

[16] Zhou P, Homberg JR, Fang Q, Wang J, Li W, Meng X, et al. (2019). Histamine-4 receptor antagonist JNJ7777120 inhibits pro-inflammatory microglia and prevents the progression of Parkinson-like pathology and behaviour in a rat model. Brain Behav Immun, 76:61-73. 10.1016/j.bbi.2018.11.006 30408497

[17] Fang QY, Xicoy H, Shen JQ, Luchetti S, Dai D, Zhou P, et al. (2021). Histamine-4 receptor antagonist ameliorates Parkinson-like pathology in the striatum. Brain Behav Immun, 92:127-138. 10.1016/j.bbi.2020.11.036 33249171

[18] Barata-Antunes S, Cristóvao AC, Pires J, Rocha SM, and Bernardino L (2017). Dual role of histamine on microglia-induced neurodegeneration. Bba-Mol Basis Dis, 1863:764-769. 10.1016/j.bbadis.2016.12.016 28057587

[19] Zhang W, Zhang X, Zhang Y, Qu C, Zhou X, and Zhang S (2020). Histamine Induces Microglia Activation and the Release of Proinflammatory Mediators in Rat Brain Via H1R or H4R. J Neuroimmune Pharmacol, 15:280-291. 10.1007/s11481-019-09887-6 31863333

[20] Saraiva C, Barata-Antunes S, Santos T, Ferreiro E, Cristóvao AC, Serra-Almeida C, et al. (2019). Histamine modulates hippocampal inflammation and neurogenesis in adult mice. Sci Rep, 9:8384. 10.1038/s41598-019-44816-w PMC6558030 31182747

[21] Chen YN, Sha HH, Wang YW, Zhou Q, Bhuiyan P, Li NN, et al. (2020). Histamine 2/3 receptor agonists alleviate perioperative neurocognitive disorders by inhibiting microglia activation through the PI3K/AKT/FoxO1 pathway in aged rats. J Neuroinflammation, 17:217 10.1186/s12974-020-01886-2 PMC7374916 32698899

[22] Saraiva M, Vieira P and O'Garra A (2020). Biology and therapeutic potential of interleukin-10. J Exp Med, 217:e20190418. 10.1084/jem.20190418 PMC7037253 31611251

[23] Stephenson J, Nutma E, van der Valk P, and Amor S (2018). Inflammation in CNS neurodegenerative diseases. Immunology, 154:204-219. 10.1111/imm.12922 PMC5980185 29513402

[24] Hakansson A, Westberg L, Nilsson S, Buervenich S, Carmine A, Holmberg B, et al. (2005). Investigation of genes coding for inflammatory components in Parkinson's disease. Mov disord, 20:569-573. 10.1002/mds.20378 15648059

[25] Kwilasz AJ, Grace PM, Serbedzija P, Maier SF, and Watkins LR (2015). The therapeutic potential of interleukin-10 in neuroimmune diseases. Neuropharmacology, 96:55-69. 10.1016/j.neuropharm.2014.10.020 PMC5144739 25446571

[26] Wang QQ, Liu YJ, and Zhou JW (2015). Neuroinflammation in Parkinson's disease and its potential as therapeutic target. Transl Neurodegener, 4:19. 10.1186/s40035-015-0042-0 PMC4603346 26464797

[27] Collins LM, Toulouse A, Connor TJ, and Nolan YM (2012). Contributions of central and systemic inflammation to the pathophysiology of Parkinson's disease. Neuropharmacology, 62:2154-2168. 10.1016/j.neuropharm.2012.01.028 22361232

[28] Jin XX, Si XL, Lei XG, Liu HF, Shao AW, and Li LF (2024). Disruption of Dopamine Homeostasis Associated with Alteration of Proteins in Synaptic Vesicles: A Putative Central Mechanism of Parkinson's Disease Pathogenesis. Aging Dis, 15:1204-1226. 10.14336/AD.2023.0821-2 PMC11081171 37815908

[29] Bido S, Nannoni M, Muggeo S, Gambarè D, Ruffini G, Bellini E, et al. (2024). Microglia-specific gene delivery inhibits neuroinflammation and neurodegeneration in a mouse model of Parkinson's disease. Sci Transl Med, 16:eadm8563. 10.1126/scitranslmed.adm8563 39167665

[30] Qi ZX, Shen KL, Peng JY, Fan XJ, Huang HW, Jiang JL, et al. (2023). Histamine bidirectionally regulates the intrinsic excitability of parvalbumin-positive neurons in the lateral globus pallidus and promotes motor behaviour. Br J Pharmacol, 180:1379-1407. 10.1111/bph.16010 36512485

[31] Paxinos G, and Franklin KBJ (2019). The mouse brain in stereotaxic coordinates, 5th Edn. San Diego, CA: Academic Press/Elsevier.

[32] Peng JY, Shen KL, Fan XJ, Qi ZX, Huang HW, Jiang JL, et al. (2023). Receptor and Ionic Mechanism of Histamine on Mouse Dorsolateral Striatal Neurons. Mol Neurobiol, 60:183-202. 10.1007/s12035-022-03076-y 36245064

[33] Cardoso A, Martins AC, Maceiras AR, Liu W, Castro I, Castro AG, et al. (2021). Interleukin-10 induces interferon-γ-dependent emergency myelopoiesis. Cell Rep, 37:109887. 10.1016/j.celrep.2021.109887 34706233

[34] Jiang R, Lu ZC, Wang CX, Xiao J, Liu QQ, Xu XD, et al. (2024). Beta2 adrenergic receptor-mediated abnormal myelopoiesis drives neuroinflammation in aged patients with traumatic brain injury. Sci Adv, 10:eadp5239. 10.1126/sciadv.adp5239 PMC11259178 39028822

[35] Zhao Z, Li FY, Ning JW, Peng R, Shang JM, Liu H, et al. (2021). Novel compound FLZ alleviates rotenoneinduced PD mouse model by suppressing TLR4/MyD88/NF-κB pathway through microbiotaegutebrain axis. Acta Pharm Sin B, 11:2859-2879. 10.1016/j.apsb.2021.03.020 PMC8463266 34589401

[36] Ma XY, Shin YJ, Yoo JW, Park HS, and Kim DH (2023). Extracellular vesicles derived from Porphyromonas gingivalis induce trigeminal nerve-mediated cognitive impairment. J Adv Res, 54:293-303. 10.1016/j.jare.2023.02.006 PMC10703712 36796586

[37] Bouet V, Boulouard M, Toutain J, Divoux D, Bernaudin M, Schumann-Bard P, et al. (2009). The adhesive removal test: a sensitive method to assess sensorimotor deficits in mice. Nature Protocols, 4:1560-1564. 10.1038/nprot.2009.125 19798088

[38] Fleming SM, Salcedo J, Fernagut PO, Rockenstein E, Masliah E, Levine MS, et al. (2004). Early and progressive sensorimotor anomalies in mice overexpressing wild-type human alpha-synuclein. J Neurosci, 24:9434-9440. 10.1523/JNEUROSCI.3080-04.2004 PMC6730110 15496679

[39] Glajch KE, Fleming SM, Surmeier DJ, and Osten P (2012). Sensorimotor assessment of the unilateral 6-hydroxydopamine mouse model of Parkinson's disease. Behav Brain Res, 230:309-316. 10.1016/j.bbr.2011.12.007 PMC3324279 22178078

[40] Iida T, Yoshikawa T, Matsuzawa T, Naganuma F, Nakamura T, Miura Y, et al. (2015). Histamine H3 receptor in primary mouse microglia inhibits chemotaxis, phagocytosis, and cytokine secretion. Glia, 63:1213-1225. 10.1002/glia.22812 25754956

[41] Katoh Y, Niimi M, Yamamoto Y, Kawamura T, Morimoto-Ishizuka T, Sawada M, et al. (2001). Histamine production by cultured microglial cells of the mouse. Neurosci Lett, 305:181-184. 10.1016/s0304-3940(01)01835-3 11403935

[42] Porro C, Cianciulli A, and Panaro MA (2020). The Regulatory Role of IL-10 in Neurodegenerative Diseases. Biomolecules, 10. 10.3390/biom10071017 PMC7407888 32659950

[43] Sun Y, Ma JJ, Li DF, Li PG, Zhou XL, Li Y, et al. (2019). Interleukin-10 inhibits interleukin-1β production and inflammasome activation of microglia in epileptic seizures. J Neuroinflamm, 16:66. 10.1186/s12974-019-1452-1 PMC6437919 30922332

[44] Branco A, Yoshikawa FSY, Pietrobon AJ, and Sato MN (2018). Role of Histamine in Modulating the Immune Response and Inflammation. Mediators Inflamm, 2018:9524075. 10.1155/2018/9524075 PMC6129797 30224900

[45] Ferreira R, Santos T, Gonçalves J, Baltazar G, Ferreira L, Agasse F, et al. (2012). Histamine modulates microglia function. J Neuroinflamm, 9:90. 10.1186/1742-2094-9-90 PMC3583187 22569158

[46] Ernst O, Glucksam-Galnoy Y, Bhatta B, Athamna M, Ben-Dror I, Glick Y, et al. (2019). Exclusive Temporal Stimulation of IL-10 Expression in LPS-Stimulated Mouse Macrophages by cAMP Inducers and Type I Interferons. Front Immunol, 10:1788. 10.3389/fimmu.2019.01788 PMC6691811 31447835

[47] Haas HL, Sergeeva OA, and Selbach O (2008). Histamine in the nervous system. Physiol Rev, 88:1183-1241. 10.1152/physrev.00043.2007 18626069

[48] Onizuka S, Tamura R, Yonaha T, Oda N, Kawasaki Y, Shirasaka T, et al. (2012). Clinical dose of lidocaine destroys the cell membrane and induces both necrosis and apoptosis in an identified Lymnaea neuron. J Anesth, 26:54-61. 10.1007/s00540-011-1260-y 22038615

[49] Wang YP, Gao WQ, Shi XY, Ding JJ, Liu W, He HB, et al. (2017). Chemotherapy drugs induce pyroptosis through caspase-3 cleavage of a gasdermin. Nature, 547:99-103. 10.1038/nature22393 28459430

[50] Karpenko MN, Vasilishina AA, Gromova EA, Muruzheva ZM, and Bernadotte A (2018). Interleukin-1β, interleukin-1 receptor antagonist, interleukin-6, interleukin-10, and tumor necrosis factor-α levels in CSF and serum in relation to the clinical diversity of Parkinson's disease. Cell Immunol, 334:99-99. 10.1016/j.cellimm.2018.02.011 29478949

[51] Kempuraj D, Thangavel R, Natteru PA, Selvakumar GP, Saeed D, Zahoor H, et al. (2016). Neuroinflammation Induces Neurodegeneration. J Neurol Neurosurg Spine, 1:1003. PMC5260818 28127589

[52] Huang Q, Wang YF, Chen SS, and Liang FX (2024). Glycometabolic Reprogramming of Microglia in Neurodegenerative Diseases: Insights from Neuroinflammation. Aging Dis, 15:1155-1175. 10.14336/AD.2023.0807 PMC11081147 37611905

[53] Bernardino L (2022). Histamine in the crosstalk between innate immune cells and neurons: Relevance for brain homeostasis and disease. Curr Top Behav Neurosci, 59:261-288. 10.1007/7854_2021_235 34432259

[54] Hu W, and Chen Z (2017). The roles of histamine and its receptor ligands in central nervous system disorders: An update. Pharmacol Ther, 175:116-132. 10.1016/j.pharmthera.2017.02.039 28223162

[55] Grace PM, Loram LC, Christianson JP, Strand KA, Flyer-Adams JG, Penzkover KR, et al. (2017). Behavioral assessment of neuropathic pain, fatigue, and anxiety in experimental autoimmune encephalomyelitis (EAE) and attenuation by interleukin-10 gene therapy. Brain Behav Immun, 59:49-54. 10.1016/j.bbi.2016.05.012 PMC5108696 27189037

[56] Tylutka A, Zabinski P, Walas L, and Zembron-Lacny A (2024). Neuroinflammation as a Link in Parkinson's and Alzheimer's Diseases: A Systematic Review and MetaAnalysis. Aging Dis, doi: 10.14336/AD.2024.1174. PMC12539528 39751856

[57] Gravel M, Béland LC, Soucy G, Abdelhamid E, Rahimian R, Gravel C, et al. (2016). IL-10 Controls Early Microglial Phenotypes and Disease Onset in ALS Caused by Misfolded Superoxide Dismutase 1. J Neurosci, 36:1031-1048. 10.1523/JNEUROSCI.0854-15.2016 PMC6601999 26791230

[58] Tao WH, Zhang GB, Liu CY, Jin LD, Li XH, and Yang SF (2023). Low-dose LPS alleviates early brain injury after SAH by modulating microglial M1/M2 polarization via USP19/FOXO1/IL-10/IL-10R1 signaling. Redox Biol, 66:102863. 10.1016/j.redox.2023.102863 PMC10494318 37672892

[59] Shan YF, Gao YN, Zhang L, Ma LL, Shi YW, and Liu X (2019). H4 Receptor Inhibits Lipopolysaccharide-induced NF-kappa B Activation by Interacting with Tumor Necrosis Factor Receptor-Associated Factor 6. Neuroscience, 398:113-125. 10.1016/j.neuroscience.2018.11.050 30528857

[60] Wang JG, Liu B, Sun FJ, Xu Y, Luan HY, Yang MZ, et al. (2022). Histamine H3R antagonist counteracts the impaired hippocampal neurogenesis in Lipopolysaccharide-induced neuroinflammation. Int Immunopharmacol, 110:109045. 10.1016/j.intimp.2022.109045 35978505

[61] del Rio R, Noubade R, Saligrama N, Wall EH, Krementsov DN, Poynter ME, et al. (2012). Histamine H4 receptor optimizes T regulatory cell frequency and facilitates anti-inflammatory responses within the central nervous system. J Immunol, 188:541-547. 10.4049/jimmunol.1101498 PMC3253209 22147765

[62] Flamand N, Plante H, Picard S, Laviolette M, and Borgeat P (2004). Histamine-induced inhibition of leukotriene biosynthesis in human neutrophils:: involvement of the H2 receptor and cAMP. Brit J Pharmacol, 141:552-561. 10.1038/sj.bjp.0705654 PMC1574237 14744809

[63] Wu HY, Tang XQ, Mao XF, and Wang YX (2017). Autocrine Interleukin-10 Mediates Glucagon-Like Peptide-1 Receptor-Induced Spinal Microglial beta-Endorphin Expression. J Neurosci, 37:11701-11714. 10.1523/JNEUROSCI.1799-17.2017 PMC6705741 29084866

[64] Elenkov IJ, Webster E, Papanicolaou DA, Fleisher TA, Chrousos GP, and Wilder RL (1998). Histamine potently suppresses human IL-12 and stimulates IL-10 production via H2 receptors. J Immunol, 161:2586-2593. 9725260

[65] Akanda N, and Elinder F (2006). Biophysical properties of the apoptosis-inducing plasma membrane voltage-dependent anion channel. Biophys J, 90:4405-4417. 10.1529/biophysj.105.080028 PMC1471872 16581845

[66] Sribnick EA, Ray SK, Nowak MW, Li L, and Banik NL (2004). 17β-estradiol attenuates glutamate-induced apoptosis and preserves electrophysiologic function in primary cortical neurons. J Neurosci Res, 76:688-696. 10.1002/jnr.20124 15139027

[67] Elinder F, Akanda N, Tofighi R, Shimizu S, Tsujimoto Y, Orrenius S, et al. (2005). Opening of plasma membrane voltage-dependent anion channels (VDAC) precedes caspase activation in neuronal apoptosis induced by toxic stimuli. Cell Death Differ, 12:1134-1140. 10.1038/sj.cdd.4401646 15861186

